# Integrated In Silico Prioritization of Antidiabetic Phytochemicals from *Uvaria chamae* P. Beauv. Based on Docking, Induced-Fit Docking, QSAR, and ADMET Analyses

**DOI:** 10.3390/molecules31111879

**Published:** 2026-05-29

**Authors:** Toussaint Sovegnon, Sèdami Medegan Fagla, Brice Boris Legba, Joseph Lorent, Joelle Quetin-Leclercq, Habib Ganfon, Jean-Robert Klotoe, Fernand Gbaguidi, Victorien Dougnon

**Affiliations:** 1 Research Unit in Applied Microbiology and Pharmacology of Natural Substances, Research Laboratory in Applied Biology, Polytechnic School of Abomey-Calavi, University of Abomey-Calavi, Abomey Calavi P.O. Box 526, Benin; semevo.sovegnon@uclouvain.be (T.S.); legba.boris5@gmail.com (B.B.L.); jrklotoe@yahoo.fr (J.-R.K.); 2 Department of Cellular and Molecular Pharmacology, Louvain Drug Research Institute, UCLouvain, Avenue E. Mounier, 73, B1.73.05, 1200 Brussels, Belgium; joseph.lorent@uclouvain.be; 3 Laboratory of Pharmacognosy, Faculty of Health Sciences, University of Abomey-Calavi, 01, Cotonou P.O. Box 188, Benin; habib.ganfon@uac.bj; 4 Medicinal and Organic Chemistry Laboratory, Faculty of Health Sciences, University of Abomey-Calavi, 01, Cotonou P.O. Box 188, Benin; smedeganfagla@presidence.bj; 5 Pharmacognosy Research Group, Louvain Drug Research Institute, UCLouvain, Avenue E. Mounier, 72, B1.72.03, 1200 Brussels, Belgium; joelle.leclercq@uclouvain.be; 6 National Laboratory of Pharmacognosy, Beninese Center for Scientific and Technical Research, 01, Oganla, Porto-Novo P.O. Box 06, Benin; ahokannou@yahoo.fr

**Keywords:** *Uvaria chamae* P. Beauv., type 2 diabetes, medicinal plants, polypharmacological mixture, molecular docking, PPARγ, PTP1B, ADMET

## Abstract

Background: Diabetes mellitus remains a major public health concern, particularly in sub-Saharan Africa where type 2 diabetes predominates. In West Africa, *Uvaria chamae* P. Beauv. is traditionally used for diabetes management. This study investigates previously reported metabolites from *Uvaria chamae* using an integrated in silico approach to explore their potential antidiabetic activity and underlying mechanisms. Methods: A comprehensive literature survey identified 106 phytochemicals from stems, roots, leaves, and seeds. Diabetes-related protein targets were retrieved from the RCSB Protein Data Bank, while ligand structures were obtained from PubChem and the COCONUT database. Molecular docking, MM-GBSA rescoring, induced-fit docking, QSAR, and ADMET analyses were performed to evaluate interaction profiles, predicted activity, and developability. Results: The integrated analysis supports a polypharmacological mixture-based profile with organ-associated trends. Stem- and root-derived flavonoids, particularly isouvaretin and diuvaretin, showed the most consistent profiles for PPARγ-related pathways, while uvarinol was associated with PTP1B. Leaf alkaloids were mainly linked to DPP-4 and digestive enzyme inhibition. These compounds displayed more favorable predicted pharmacokinetic and toxicity profiles compared to acetogenins, which, despite favorable binding energies, were not prioritized as drug-like candidates due to their high lipophilicity, low QED values, and predicted toxicity liabilities, but may contribute to extract-level activity. Conclusion: These findings provide a hypothesis-generating and hierarchical framework for the prioritization of *Uvaria chamae* metabolites and extracts, supporting further experimental validation through enzymatic, cellular, and gene expression studies.

## 1. Introduction

Diabetes mellitus encompasses a group of chronic metabolic disorders characterized by persistent hyperglycemia resulting from insufficient insulin secretion, insulin resistance, or a combination of these two abnormalities. This disruption leads to decreased cellular glucose utilization and an inappropriate increase in hepatic glucose production through gluconeogenesis and glycogenolysis [1,2]. Due to its increasing prevalence worldwide, in both developed and developing countries, diabetes represents a major public health challenge [1,3]. Among the various forms of diabetes, type 2 diabetes (T2D) remains the most prevalent, particularly among middle-aged and older adults [1,4]. Diabetes-related morbidity and mortality are largely attributable to its chronic complications, classified as microvascular and macrovascular complications [5]. The former, such as diabetic retinopathy, nephropathy and neuropathy, affect small blood vessels and cause progressive tissue damage, while the latter affect large arteries and represent the main cause of death in diabetic patients, notably through cardiovascular diseases, cerebrovascular diseases and peripheral arteriopathy [6]. The conventional management of type 2 diabetes relies on lifestyle modifications, dietary control, and pharmacological interventions such as oral hypoglycemic agents or insulin therapy [1,3]. Advances in rational drug design have fostered the development of numerous synthetic antidiabetic agents, particularly through in silico approaches, which allow the identification of biomolecules capable of interacting specifically with molecular targets involved in human metabolic pathways [7,8]. Currently, the pharmacological treatment of diabetes relies on several therapeutic classes, including biguanides, dipeptidyl peptidase-4 (DPP-4) inhibitors, sodium-glucose cotransporter 2 (SGLT2) inhibitors, sulfonylureas, and thiazolidinediones [1,2,8]. Among these options, metformin, the main representative of the biguanides, has historically remained the first-line treatment for type 2 diabetes and has been used in clinical practice since the 1960s [2,9]. It improves insulin sensitivity and contributes to blood glucose control, with a low risk of hypoglycemia when used as monotherapy and without promoting significant weight gain [2,9]. However, despite the diversity of available therapeutic options, the persistence of adverse effects, limitations in efficacy for some patients, and economic and accessibility constraints highlight the need to search for new antidiabetic alternatives [2].

From this perspective, medicinal plants remain a valuable source of bioactive compounds with antihyperglycemic, antioxidant, and anti-inflammatory properties, which can complement or serve as an alternative to conventional therapies [3]. Among them, *Uvaria chamae* P. Beauv., a medicinal plant from the tropical forests of West and Central Africa, is recognized for various pharmacological properties, including antiparasitic, antidiarrheal, antimicrobial, anti-inflammatory, antioxidant, and antidiabetic effects [10,11,12,13,14,15]. The works of Emordi et al. [16,17] have demonstrated the antidiabetic and lipid-lowering effects of hydroethanolic and ethanolic extracts of *Uvaria chamae* P. Beauv roots in diabetic albino rats. These studies showed significant reductions (*p* < 0.05) in body weight, plasma glucose, cholesterol, and low-density lipoprotein (LDL) compared to the control group. At doses of 100, 250, and 400 mg/kg body weight, the extract induced a marked reduction in blood glucose, associated with the histological presence of islets of Langerhans of varying sizes surrounded by normal-appearing exocrine tissue. Similarly, Sanvee et al. (2024) [18] reported strong antihyperglycemic activity of the hydroethanolic leaf extract (*p* < 0.05), associated with a high content of phenolic compounds (147.93 ± 1.01 mg/g). However, although these results support the antidiabetic potential of the species, most available work still focuses on crude extracts, while few studies have investigated the isolation and identification of the bioactive molecules specifically involved in this effect. Furthermore, the synthesis work of Agbebi et al. [19] and Abu et al. [11] shows that *Uvaria chamae* P. Beauv is a species particularly rich in secondary metabolites, distributed throughout different plant organs, including the roots, stems, leaves, seeds. The main chemical families already reported in *Uvaria chamae* include flavonoids, alkaloids, essential oils, and annonaceous acetogenins, several of which have been associated with the modulation of carbohydrate-digesting enzymes and insulin-related pathways, particularly flavonoids and alkaloids [11,19,20,21,22]. However, despite this phytochemical diversity and the various biological effects described for the species, the exploration of the compounds specifically involved in antidiabetic activity, as well as the understanding of their mechanisms of action, remains limited. In this context, in silico approaches appear as strategic tools for the rapid screening of bioactive compounds, the prediction of their interactions with relevant targets, and the rational guidance of subsequent experimental steps, particularly in the exploration of natural substances for therapeutic purposes [7]. The present study was undertaken to integrate an exploratory investigation of biomolecules previously reported in *Uvaria chamae* P. Beauv. with a mechanistic assessment of their potential interactions with key molecular targets implicated in diabetes pathophysiology, including PPARγ, PTP1B, SIRT6, DPP-4, α-amylase, and maltase-glucoamylase. More specifically, the study sought to compile the molecules identified or isolated from the different organs of *Uvaria chamae* P.Beauv, to characterize, using in silico approaches, their molecular interactions with major diabetes-related targets in order to gain insight into their possible mechanisms of action, and to predict the ADMET properties of the selected compounds, with particular emphasis on safety, bioavailability, and pharmacokinetic behavior.

## 2. Results

Virtual screening, molecular docking, MM-GBSA analysis and induced fit docking of compounds targeting proteins involved in diabetes.

**Figure 1:**
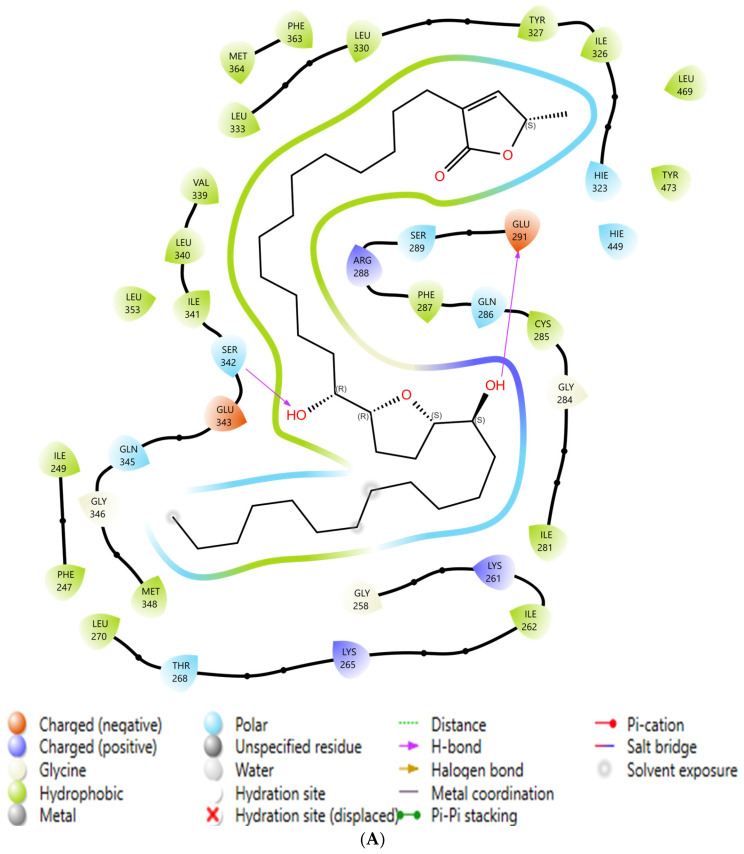

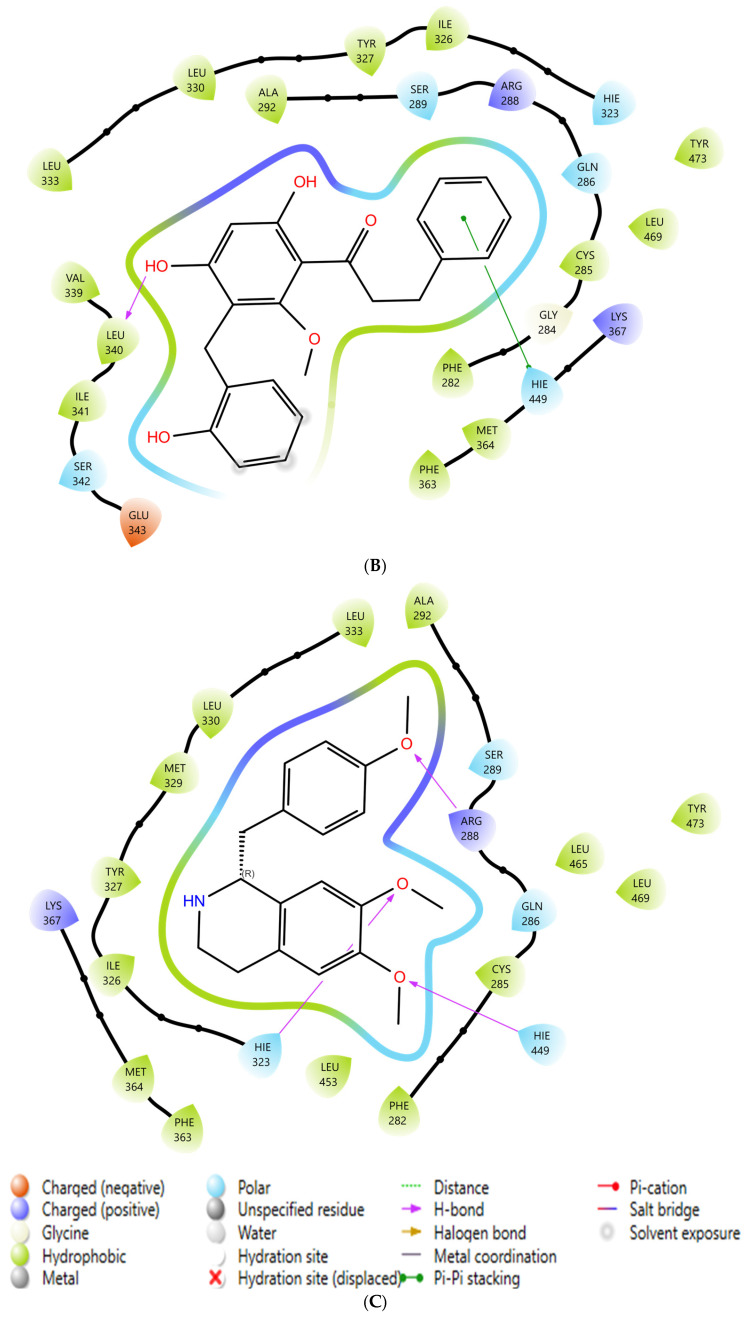

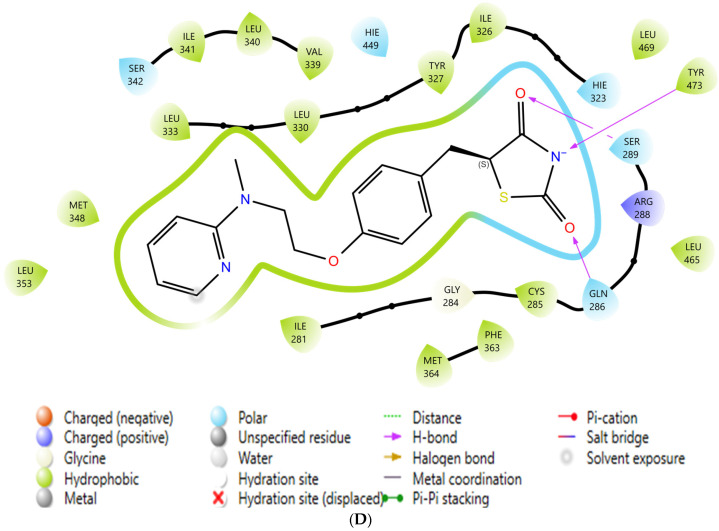
Two-dimensional ligand–protein interaction maps of selected compounds with PPARγ (PDB ID: 2PRG). (**A**) Solamin interaction; (**B**) Isouvaretin interaction; (**C**) O,O-Dimethylcoclaurine interaction; (**D**) Rosiglitazone (standard) interaction.

Structural analysis combining molecular docking, MM-GBSA binding free energy estimation, and induced fit docking (IFD) revealed distinct interaction profiles among the major secondary metabolites of *Uvaria chamae* across the investigated diabetes-related targets. Detailed energetic values are summarized in Table 1 and Table 2. Within the insulin-sensitizing axis, flavonoids such as isouvaretin and diuvaretin, identified from stems and roots, exhibited the most favorable interaction profiles across both PPARγ structures (2Q5S and 2PRG) (Figure 1 and Figure S3). Their binding involved hydrogen bonds with residues such as SER342 and SER289, along with additional interactions involving LEU340 and ARG288. In comparison, solamin, a root-derived acetogenin, displayed an initial interaction profile but no stable induced-fit pose was retained, while the leaf alkaloid O,O-dimethylcoclaurin showed a more moderate interaction pattern. The reference ligands remained among the strongest binders across both structures. Extending this analysis to insulin signaling, PTP1B (2QBQ) showed a distinct interaction pattern. The flavonoid uvarinol, isolated from stems and roots, exhibited the most favorable profile among the natural ligands, with a hydrogen bond involving TYR46, π–π stacking with PHE182, and a π–cation interaction with ARG45, together with hydrophobic contacts involving residues such as VAL49, LEU88, CYS215, and ILE219. Root acetogenins, including cis-uvariamicin and squamocin, also showed relevant interaction profiles, although with lower stability after induced fit docking compared to uvarinol. The reference ligand remained stronger overall. A related interaction pattern was observed for digestive enzymes. In α-amylase (3L2M), the flavonoid diuvaretin (stems and roots) exhibited the most favorable profile among natural compounds, forming hydrogen bonds with VAL163 and GLN63, while root acetogenins such as uvariamicin II and annotemoyin-1 also showed relevant interactions. In the human α-amylase structure (1B2Y), the leaf alkaloid (+)-armepavine formed hydrogen bonds with TYR151 and ASP300, along with salt-bridge interactions involving GLU233 and ASP300 and a π–cation interaction with TYR62. In maltase-glucoamylase (2QMJ), the leaf alkaloid corydine exhibited a distinct interaction pattern involving hydrophobic contacts with PHE450, TRP406, TYR299, and PHE575, together with a salt bridge involving ASP542 and π–π interactions with PHE575. Reference inhibitors remained stronger across these targets. In the incretin-related pathway, DPP-4 (3C45) interactions were dominated by leaf alkaloids. Nornanternine formed hydrogen bonds with GLU205, GLU206, and TYR547, together with electrostatic interactions within the catalytic site. Similarly, the leaf alkaloid (+)-armepavine showed a consistent interaction pattern, with interactions preserved after induced fit docking. The reference ligand remained stronger after structural refinement. Finally, SIRT6 (3K35) interactions were mainly associated with root-derived acetogenins. Cis-uvariamicin I, uvariamicin II, and uvariamicin-I displayed interaction profiles involving hydrogen bonds with residues such as HIS131, GLN111, LYS13, and ALA51, together with extensive hydrophobic contacts. The reference ligand showed stronger binding overall (Table 1 and Table 2).

### 2.1. Biological Activities of Hit Compounds

The predicted biological potency values, expressed as pIC_50_ (Table 3), indicate variable activity levels depending on both the target and the chemical nature of the compounds. In general, compounds with pIC_50_ values close to or above 6 may be considered biologically relevant, with submicromolar to low micromolar activity, whereas values around 5 suggest moderate activity. Concerning the nuclear receptor PPARγ (2Q5S), flavonoids derived from stems and roots appear to be the most active studied ligands. Isouvaretin and diuvaretin exhibited predicted pIC_50_ values of 6.224 and 6.204, respectively, which are close to those of the reference ligands NZA (6.317) and rosiglitazone (6.417). These results support a plausible modulation of the receptor and are consistent with a potential insulin-sensitizing mechanism. By contrast, the root acetogenin solamine showed a lower predicted pIC_50_ (5.009), suggesting a weaker contribution to this pathway. The leaf alkaloid O,O-dimethylcoclaurine displayed an intermediate but still relevant pIC_50_ (6.169), indicating that foliar alkaloids may also contribute, although to a lesser extent, to PPARγ-related effects. With respect to protein tyrosine phosphatase 1B (2QBQ), the best interating compounds displayed relatively close predicted activities. Cis-uvariamicin and squamocin both showed a pIC_50_ of 5.556, whereas uvarinol reached 5.426. These values are comparable to, and in fact slightly higher than that of the reference ligand (5.219), suggesting a plausible moderate inhibition of the enzyme. Such an effect would be consistent with an improvement in insulin signaling, and the similarity of the predicted values supports the idea of a shared contribution by several compounds, particularly those derived from roots. For DPP-4 (3C45), leaf alkaloids clearly exhibited the most favorable predicted activities. (+)-Armepavine showed a pIC_50_ of 6.557, indicating substantial inhibitory potential, while nornanternine reached 5.925, consistent with moderate but still relevant activity. Although both values remain below that of the reference ligand (7.100), they nevertheless support a biologically meaningful contribution of foliar alkaloids to the modulation of the incretin pathway. Regarding maltase-glucoamylase (2QMJ), the leaf alkaloid corydine exhibited a predicted pIC_50_ of 4.960, which is higher than that of the reference inhibitor α-acarbose (4.123). Although this value remains within the moderate activity range, it suggests a plausible inhibitory effect on this digestive enzyme and supports the involvement of foliar alkaloids in the modulation of carbohydrate digestion and postprandial glucose release. Overall, the QSAR data reveal a clear target-dependent distribution of predicted biological activities. Stem- and root-derived flavonoids, especially isouvaretin and diuvaretin, showed the strongest predicted activity toward PPARγ. Leaf alkaloids, particularly (+)-armepavine, nornanternine, and corydine, were more strongly associated with DPP-4 and 2QMJ, supporting their potential role in the incretin pathway and carbohydrate digestion. Root-derived compounds, including cis-uvariamicin, squamocin, and uvarinol, contributed primarily to the predicted inhibition of PTP1B.

### 2.2. In Silico Prediction of the ADMET Properties of Identified Inhibitors

Oral absorption

The predicted absorption profiles revealed a heterogeneous but informative pattern across the selected natural compounds and pharmacologically relevant reference ligands. Caco-2 permeability, predicted using the Caco-2 human colorectal adenocarcinoma cell model commonly employed as an in vitro surrogate of intestinal absorption, was within or close to the favorable range for Isouvaretin (−4.956), Diuvaretin (−5.045), Solamin (−5.103), O,O-dimethylcoclaurine (−5.030), cis-Uvariamicin I (−5.138), Uvarinol (−5.126), Nornanternine (−4.991), (+)-Armepavine (−4.494), Uvariamicin II (−5.070), Uvariamicin-I (−5.067), Annotemoyin-1 (−5.064), and Corydine (−5.012), whereas Squamocin (−5.184) was slightly below this threshold. According to ADMETlab, Caco-2 values above −5.15 log cm/s are generally considered favorable. Among the reference ligands, Rosiglitazone (−4.986) and 5-chloro-1-(4-chloroben-zyl)-3-(phenylthio)-1H-indole-2-carboxylic acid (−4.816) also showed favorable predicted Caco-2 permeability, whereas the DPP-4 reference ligand, (2S,3S)-3-{3-[2-chloro-4-(methylsulfonyl)phenyl]-1,2,4-oxadiazol-5-yl}-1-cyclopentylidene-4-cyclopropyl-1-fluorobutan-2-amine, showed a slightly lower but still moderate value (−5.300). In contrast, α-acarbose (−7.048), ADP-ribose (−6.023), and α-cyclodextrin (−8.280) displayed very poor predicted permeability, consistent with poorly permeant or predominantly luminal behavior [23].

The corresponding MDCK permeability values, predicted using the MDCK (Madin–Darby Canine Kidney) epithelial cell model, were broadly consistent with the Caco-2 results and suggested a similar overall tendency for passive membrane permeation [24]. Most compounds remained within an intermediate MDCK permeability range, including Isouvaretin (−4.763), Diuvaretin (−4.743), Solamin (−4.849), O,O-dimethylcoclaurine (−4.764), cis-Uvariamicin I (−4.836), Uvarinol (−4.800), Squamocin (−4.766), Nornanternine (−4.789), Uvariamicin II (−4.893), Uvariamicin-I (−4.878), Annotemoyin-1 (−4.861), and Corydine (−4.770), whereas (+)-Armepavine (−4.621) and Rosiglitazone (−4.506) showed comparatively more favorable MDCK profiles. In ADMETlab, MDCK interpretation is linked to the apparent permeability coefficient (Papp), with low permeability below 2 × 10^−6^ cm/s, intermediate permeability between 2 and 20 × 10^−6^ cm/s, and high permeability above 20 × 10^−6^ cm/s.

Predictions related to P-gp, i.e., P-glycoprotein (also known as ABCB1 or MDR1), an ATP-dependent efflux transporter that can reduce intestinal absorption, suggested substantial differences in efflux liability among the tested molecules [23]. Isouvaretin, Diuvaretin, O,O-dimethylcoclaurine, (+)-Armepavine, Rosiglitazone, and 5-chloro-1-(4-chloroben-zyl)-3-(phenylthio)-1H-indole-2-carboxylic acid were predicted as P-gp inhibitors, whereas Squamocin and α-acarbose were predicted as strong P-gp substrates, suggesting a higher risk of efflux-limited absorption. Solamin and O,O-dimethylcoclaurine also showed some substrate tendency, while cis-Uvariamicin I, Uvarinol, Uvariamicin II, Uvariamicin-I, Annotemoyin-1, Corydine, Rosiglitazone, and the PTP1B reference ligand were predicted as non-substrates. (+)-Armepavine displayed an intermediate substrate tendency.

The predictions for HIA (Human Intestinal Absorption) and oral bioavailability classes should be interpreted cautiously. In ADMETlab, these outputs are class-probability predictions rather than direct experimental measurements. HIA+ corresponds to the poorly absorbed class (<30% intestinal absorption), whereas F20+, F30+, and F50+ indicate a higher probability of belonging to the low oral bioavailability classes below 20%, 30%, and 50%, respectively [23]. Under this interpretation, Solamin, cis-Uvariamicin I, Uvariamicin II, Uvariamicin-I, and Annotemoyin-1 showed the most favorable overall profiles across HIA and oral-bioavailability-related outputs, whereas Isouvaretin, Diuvaretin, Nornanternine, (+)-Armepavine, and the highly hydrophilic standards displayed less favorable absorption-related patterns. Uvarinol and Corydine showed intermediate profiles, while Rosiglitazone and 5-chloro-1-(4-chloroben-zyl)-3-(phenylthio)-1H-indole-2-carboxylic acid combined favorable membrane permeability with relatively low HIA and bioavailability-related probabilities. Overall, these data support a mixed absorption pattern, combining moderately permeable compounds with plausible systemic exposure and poorly permeable molecules more likely to contribute to local intestinal effects.

Distribution

Predicted distribution parameters revealed marked differences in plasma binding and tissue disposition across both the selected natural compounds and the reference ligands. Plasma protein binding (PPB) was predicted to be high for most flavonoids and acetogenins, including Isouvaretin (98.2%), Diuvaretin (98.1%), Uvarinol (98.3%), Solamin (100.2%), cis-Uvariamicin I (99.6%), Squamocin (95.8%), Uvariamicin II (100.7%), Uvariamicin-I (100.7%), and Annotemoyin-1 (100.0%). A similarly high binding profile was observed for the reference ligands 4-bromo-3-(carboxymethoxy)-5-{3-[(3,3,5,5-tetramethylcyclohexyl)amino]phenyl}thiophene-2-carboxylic acid (98.7%), Rosiglitazone (99.5%), and 5-chloro-1-(4-chloroben-zyl)-3-(phenylthio)-1H-indole-2-carboxylic acid (99.3%), whereas the reference ligand (2S,3S)-3-{3-[2-chloro-4-(methylsulfonyl)phenyl]-1,2,4-oxadiazol-5-yl}-1-cyclopentylidene-4-cyclopropyl-1-fluorobutan-2-amine showed a lower PPB (88.0%). By contrast, several alkaloids displayed lower predicted PPB, including O,O-dimethylcoclaurine (64.1%), Nornanternine (66.2%), Corydine (78.9%), and especially (+)-Armepavine (49.3%), suggesting comparatively larger unbound fractions. Very low predicted binding was observed for α-acarbose (15.9%), ADP-ribose (27.2%), and α-cyclodextrin.

The predicted volume of distribution (VD) further differentiated these profiles. Solamine showed a markedly elevated VD (29.422), suggesting extensive tissue distribution, whereas Isouvaretin (2.387), Uvarinol (2.168), (+)-Armepavine (1.532), Uvariamicin II (1.641), Uvariamicin-I (1.662), Annotemoyin-1 (1.481), cis-Uvariamicin I (1.399), and Corydine (1.180) remained compatible with appreciable tissue penetration. The reference ligands 4-bromo-3-(carboxymethoxy)-5-{3-[(3,3,5,5-tetramethylcyclohexyl)amino]phenyl}thiophene-2-carboxylic acid (0.646), Rosiglitazone (0.235), and 5-chloro-1-(4-chloroben-zyl)-3-(phenylthio)-1H-indole-2-carboxylic acid (0.617) showed more limited predicted distribution, while O,O-dimethylcoclaurine (0.103), Diuvaretin (0.384), Squamocin (0.372), Nornanternine (0.476), the reference ligand (2S,3S)-3-{3-[2-chloro-4-(methylsulfonyl)phenyl]-1,2,4-oxadiazol-5-yl}-1-cyclopentylidene-4-cyclopropyl-1-fluorobutan-2-amine (0.031), α-acarbose (−0.527), ADP-ribose (−0.479), and α-cyclodextrin (−0.451) displayed the most restricted predicted distribution.

Blood–brain barrier (BBB) penetration was predicted to be absent or weak for nearly all natural compounds, including Isouvaretin, Diuvaretin, Solamin, O,O-dimethylcoclaurine, cis-Uvariamicin I, Uvarinol, Squamocin, Uvariamicin II, Uvariamicine-I, Annotemoyin-1, and Corydine, whereas Nornanternine and (+)-Armepavine showed only marginal BBB probability. Among the reference ligands, Rosiglitazone was the only compound strongly predicted as BBB-permeant, while 4-bromo-3-(carboxymethoxy)-5-{3-[(3,3,5,5-tetramethylcyclohexyl)amino]phenyl}thiophene-2-carboxylic acid, 5-chloro-1-(4-chloroben-zyl)-3-(phenylthio)-1H-indole-2-carboxylic acid, α-acarbose, ADP-ribose, and α-cyclodextrin remained non-penetrant. Overall, these data support a predominantly peripheral distribution pattern for the natural metabolites, with notable differences in free fraction and tissue disposition across chemical families.

Metabolism

Predicted interactions with cytochrome P450 (CYP) isoforms revealed marked family-dependent differences in metabolic liability. Among the flavonoids, Isouvaretin and Diuvaretin showed the most interaction-prone profiles. Both were predicted as CYP2C9 inhibitors and substrates, while Isouvaretin was also predicted as a CYP1A2 inhibitor and substrate. Diuvaretin further showed a strong probability of CYP2C19 inhibition and CYP3A4 substrate behavior. Uvarinol displayed a distinct profile characterized by predicted inhibition of CYP2C19 and CYP2C9, together with substrate behavior toward CYP2C9 and CYP2D6, but little evidence of CYP1A2 or CYP3A4 involvement.

The alkaloids were generally characterized by broader substrate than inhibitor behavior. O,O-dimethylcoclaurine showed predicted substrate liability for CYP1A2, CYP2C19, CYP2C9, CYP2D6, and CYP3A4, and was also predicted as a CYP2D6 inhibitor. Nornanternine and (+)-Armepavine were predicted mainly as substrates of CYP1A2, CYP2C19, CYP2C9, and CYP2D6, with more limited inhibitory profiles, whereas Corydine combined substrate behavior toward CYP1A2, CYP2C19, CYP2C9, CYP2D6, and CYP3A4 with weaker predicted inhibition of CYP1A2.

The acetogenins showed more heterogeneous patterns. Solamin, cis-Uvariamicin I, Uvariamicin II, Uvariamicin-I, Squamocin, and Annotemoyin-1 generally exhibited limited CYP1A2 and CYP2D6 inhibition, but several retained substrate liability toward CYP2C9 and/or CYP3A4. Notably, Solamin and Uvariamicin-I were predicted as CYP2C19 inhibitors, cis-Uvariamicin I showed some CYP3A4 inhibition and substrate behavior toward CYP2C9, and Annotemoyin-1 combined CYP1A2, CYP2C19, and CYP3A4 inhibition with CYP2C9 substrate liability. Squamocin showed the lowest overall CYP interaction burden within this group.

Among the reference ligands, Rosiglitazone showed mixed CYP1A2 inhibition and CYP2C9 substrate behavior, whereas 4-bromo-3-(carboxymethoxy)-5-{3-[(3,3,5,5-tetramethylcyclohexyl)amino]phenyl}thiophene-2-carboxylic acid was predicted mainly as a CYP3A4 substrate. By contrast, 5-chloro-1-(4-chloroben-zyl)-3-(phenylthio)-1H-indole-2-carboxylic acid, α-acarbose, ADP-ribose, and α-cyclodextrin showed minimal overall CYP interaction, consistent with lower hepatic metabolic involvement.

Excretion

Predicted excretion parameters suggested overall low-to-moderate systemic elimination for most of the selected natural compounds and reference ligands. According to ADMETlab 3.0, plasma clearance (CL) values below 5 mL/min/kg indicate low clearance, values between 5 and 15 mL/min/kg indicate moderate clearance, and values above 15 mL/min/kg indicate high clearance, whereas half-life (T1/2) values below 1 h, between 1 and 3 h, and above 3 h correspond to short, intermediate, and long persistence, respectively [23]. Within this framework, Isouvaretin (CL4.851; T1/2 1.101), Uvarinol (CL4.764; T1/2 1.232), Solamine (CL4.618; T1/2 2.118), cis-Uvariamicine I (CL4.984; T1/2 2.475), Uvariamicine II (CL4.585; T1/2 2.637), Uvariamicine-I (CL4.711; T1/2 2.299), and Annotemoyin-1 (CL4.905; T1/2 1.773) showed low predicted clearance associated with intermediate half-lives, consistent with progressive but not prolonged systemic persistence.

By contrast, Diuvaretin (CL7.768; T1/2 0.912), O,O-dimethylcoclaurine (CL10.33; T1/2 1.835), Nornanternine (CL5.574; T1/2 1.767), Squamocin (CL5.254; T1/2 1.317), Corydine (CL5.338; T1/2 2.918), and especially (+)-Armepavine (CL12.003; T1/2 2.337) fell within a moderate-clearance range, indicating more dynamic elimination despite generally short-to-intermediate residence times. Among the reference ligands, 4-bromo-3-(carboxymethoxy)-5-{3-[(3,3,5,5-tetramethylcyclohexyl)amino]phenyl}thiophene-2-carboxylic acid (CL2.564; T1/2 1.258), Rosiglitazone (CL6.515; T1/2 0.928), and 5-chloro-1-(4-chloroben-zyl)-3-(phenylthio)-1H-indole-2-carboxylic acid (CL0.700; T1/2 1.455) showed low-to-moderate elimination profiles, whereas α-acarbose (CL0.005; T1/2 3.813), ADP-ribose (CL1.389; T1/2 2.371), and α-cyclodextrin (CL −2.143; T1/2 6.777) remained the least efficiently cleared compounds in the dataset. The reference ligand (2S,3S)-3-{3-[2-chloro-4-(methylsulfonyl)phenyl]-1,2,4-oxadiazol-5-yl}-1-cyclopentylidene-4-cyclopropyl-1-fluorobutan-2-amine showed intermediate behavior (CL3.397; T1/2 0.688). Overall, these predictions support mainly transient to moderate systemic exposure for the natural metabolites, with no evidence of markedly prolonged persistence among the prioritized plant-derived candidates.

Toxicity

In ADMETlab 3.0, toxicity outputs are expressed as probabilities of belonging to the positive or toxic class; therefore, values closer to 0 indicate lower predicted liability, whereas values closer to 1 indicate higher predicted toxicity risk. This applies to hERG blockade, drug-induced liver injury (DILI), AMES mutagenicity, carcinogenicity, skin sensitization, eye corrosion/irritation, and respiratory toxicity. For rat oral acute toxicity, the positive class corresponds to compounds with predicted toxic doses below 500 mg/kg [23].

Predicted toxicity profiles revealed clear family-dependent differences. Among the natural compounds, the most favorable hERG profiles were observed for the flavonoids Isouvaretin (0.126), Diuvaretin (0.103), and Uvarinol (0.115), whereas several acetogenins showed distinctly higher predicted hERG liability, including Solamine (0.676), cis-Uvariamicin I (0.866), Squamocin (0.728), Uvariamicin II (0.734), Uvariamicin-I (0.694), and Annotemoyin-1 (0.627). The alkaloids were more heterogeneous, with lower values for Corydine (0.406), intermediate values for O,O-dimethylcoclaurine (0.504) and (+)-Armepavine (0.537), and a less favorable profile for Nornanternine (0.605). Among the reference ligands, 4-bromo-3-(carboxymethoxy)-5-{3-[(3,3,5,5-tetramethylcyclohexyl)amino]phenyl}thiophene-2-carboxylic acid (0.033), Rosiglitazone (0.164), ADP-ribose (0.012), α-acarbose (0.001), and α-cyclodextrin (0.000) showed low predicted hERG liability, whereas 5-chloro-1-(4-chloroben-zyl)-3-(phenylthio)-1H-indole-2-carboxylic acid (0.707) and (2S,3S)-3-{3-[2-chloro-4-(methylsulfonyl)phenyl]-1,2,4-oxadiazol-5-yl}-1-cyclopentylidene-4-cyclopropyl-1-fluorobutan-2-amine (0.508) were less favorable.

For DILI, low predicted probabilities were observed for Isouvaretin (0.028), Diuvaretin (0.019), Uvarinol (0.053), (+)-Armepavine (0.017), cis-Uvariamicine I (0.140), Uvariamicine II (0.119), and Corydine (0.053), whereas O,O-dimethylcoclaurine (0.588), Nornanternine (0.431), Solamine (0.311), Uvariamicine-I (0.295), Squamocin (0.100), and Annotemoyin-1 (0.309) indicated less favorable hepatic safety. Notably, several reference ligands also showed high predicted DILI risk, including Rosiglitazone (0.959), 4-bromo-3-(carboxymethoxy)-5-{3-[(3,3,5,5-tetramethylcyclohexyl)amino]phenyl}thiophene-2-carboxylic acid (0.997), 5-chloro-1-(4-chloroben-zyl)-3-(phenylthio)-1H-indole-2-carboxylic acid (1.000), ADP-ribose (0.998), α-acarbose (0.756), and the oxadiazole reference ligand (0.998), showing that elevated predicted hepatotoxicity was not restricted to plant-derived metabolites.

AMES mutagenicity remained comparatively low for cis-Uvariamicine I (0.049), Uvariamicine II (0.033), Diuvaretin (0.240), Isouvaretin (0.300), Solamine (0.301), Squamocin (0.369), Uvariamicine-I (0.326), Annotemoyin-1 (0.395), Rosiglitazone (0.291), 4-bromo-3-(carboxymethoxy)-5-{3-[(3,3,5,5-tetramethylcyclohexyl)amino]phenyl}thiophene-2-carboxylic acid (0.135), 5-chloro-1-(4-chloroben-zyl)-3-(phenylthio)-1H-indole-2-carboxylic acid (0.213), and the oxadiazole reference ligand (0.129). By contrast, O,O-dimethylcoclaurine (0.693), Nornanternine (0.835), Corydine (0.656), α-acarbose (0.906), and α-cyclodextrin (1.000) were less favorable, while (+)-Armepavine remained intermediate (0.343).

For rat oral acute toxicity, lower predicted liability was observed for Uvariamicine II (0.067), Isouvaretin (0.192), Diuvaretin (0.157), cis-Uvariamicin I (0.204), Solamin (0.256), Uvarinol (0.253), Squamocin (0.228), Uvariamicin-I (0.276), Annotemoyin-1 (0.293), Rosiglitazone (0.258), 5-chloro-1-(4-chloroben-zyl)-3-(phenylthio)-1H-indole-2-carboxylic acid (0.150), ADP-ribose (0.028), and α-acarbose (0.001), whereas O,O-dimethylcoclaurine (0.376), 4-bromo-3-(carboxymethoxy)-5-{3-[(3,3,5,5-tetramethylcyclohexyl)amino]phenyl}thiophene-2-carboxylic acid (0.365), and the oxadiazole reference ligand (0.254) were intermediate, and Nornanternine (0.784), (+)-Armepavine (0.837), and Corydine (0.727) were less favorable. Carcinogenicity remained relatively low for Isouvaretin (0.216), Diuvaretin (0.150), Uvarinol (0.111), cis-Uvariamicin I (0.096), Uvariamicin II (0.185), 4-bromo-3-(carboxymethoxy)-5-{3-[(3,3,5,5-tetramethylcyclohexyl)amino]phenyl}thiophene-2-carboxylic acid (0.167), Rosiglitazone (0.266), and 5-chloro-1-(4-chloroben-zyl)-3-(phenylthio)-1H-indole-2-carboxylic acid (0.195), but was higher for Nornanternine (0.570), Solamin (0.522), Uvariamicin-I (0.520), Annotemoyin-1 (0.548), Corydine (0.755), ADP-ribose (0.411), and especially α-acarbose (0.004) and α-cyclodextrin (0.000), which in this endpoint were predicted as essentially non-carcinogenic.

Skin sensitization and respiratory toxicity were generally high for most flavonoids and acetogenins, including Isouvaretin (0.846; 0.944), Diuvaretin (0.906; 0.978), Solamin (1.000; 0.974), cis-Uvariamicin I (1.000; 0.797), Uvarinol (0.964; 0.962), Squamocin (1.000; 0.975), Uvariamicin II (1.000; 0.948), Uvariamicin-I (1.000; 0.970), and Annotemoyin-1 (1.000; 0.957). Among the alkaloids, O,O-dimethylcoclaurine (0.486; 0.683), (+)-Armepavine (0.468; 0.934), and Corydine (0.530; 0.955) showed lower skin sensitization probabilities than Nornanternine (0.905; 0.984), although respiratory toxicity remained high for most. Among the reference ligands, Rosiglitazone (0.878; 0.864), 5-chloro-1-(4-chloroben-zyl)-3-(phenylthio)-1H-indole-2-carboxylic acid (0.999; 0.623), ADP-ribose (0.999; 0.923), and α-acarbose (0.999; 0.001) showed divergent profiles across these two endpoints, whereas 4-bromo-3-(carboxymethoxy)-5-{3-[(3,3,5,5-tetramethylcyclohexyl)amino]phenyl}thiophene-2-carboxylic acid (0.324; 0.156), the oxadiazole reference ligand (0.118; 0.493), and α-cyclodextrin (1.000; 0.000) remained more variable. Eye corrosion was generally low across most compounds, while eye irritation was more heterogeneous, with high values for Isouvaretin (0.977), Diuvaretin (0.959), Uvarinol (0.961), Uvariamicin II (0.963), Uvariamicin-I (0.900), and Annotemoyin-1 (0.898), but lower values for cis-Uvariamicin I (0.269), Nornanternine (0.110), Corydine (0.172), 4-bromo-3-(carboxymethoxy)-5-{3-[(3,3,5,5-tetramethylcyclohexyl)amino]phenyl}thiophene-2-carboxylic acid (0.445), Rosiglitazone (0.058), 5-chloro-1-(4-chloroben-zyl)-3-(phenylthio)-1H-indole-2-carboxylic acid (0.218), α-acarbose (0.007), the oxadiazole reference ligand (0.009), ADP-ribose (0.290), and α-cyclodextrin (0.000).

Overall, the most favorable global safety pattern among the natural metabolites was observed for Isouvaretin, Diuvaretin, and Uvarinol, which combined low hERG and DILI probabilities with relatively acceptable mutagenicity, acute oral toxicity, and carcinogenicity profiles. By contrast, several acetogenins, particularly cis-Uvariamicin I, Squamocin, Uvariamicin II, Uvariamicin-I, and Annotemoyin-1, as well as some alkaloids such as Nornanternine and (+)-Armepavine, displayed less favorable liabilities across multiple endpoints and therefore warrant greater caution in further prioritization.

### 2.3. Drug-Likeness Potential Assessment

Drug-likeness was interpreted using complementary medicinal-chemistry descriptors. Molecular weight (MW) reflects overall molecular size and is commonly expected to remain ≤ 500 Da for oral drug-like compounds. Topological polar surface area (TPSA) estimates global polarity and hydrogen-bonding capacity; values below ~140 Å^2^ are generally considered more compatible with oral absorption, whereas values below ~90 Å^2^ are more favorable for passive membrane permeation. The partition coefficient (logP) describes intrinsic lipophilicity, with values around 1–5 usually considered compatible with oral drug-like space, while logD reflects pH-dependent distribution behavior. Hydrogen bond acceptors (nHA) and donors (nHD) are part of the classical Lipinski framework, which considers compounds more likely to be orally bioavailable when MW ≤ 500, logP ≤ 5, nHA ≤ 10, and nHD ≤ 5. The number of rotatable bonds (nRot) and the flexibility index estimate conformational freedom; excessive flexibility is generally considered unfavorable for oral developability, and compounds with more than 10 rotatable bonds are less likely to show good oral bioavailability. The quantitative estimate of drug-likeness (QED) was interpreted as attractive above 0.67, intermediate between 0.49 and 0.67, and poor below 0.34. Synthetic accessibility (SAscore) reflects the relative ease of chemical synthesis, whereas PAINS, ALARM NMR, BMS, and Chelator filters were used to identify potential assay-interference or medicinal-chemistry liabilities. Additional developability guides included the GSK rule (MW ≤ 400 and logP ≤ 4) and the Golden Triangle rule (MW200–500 and logD between −2 and 5) [23,25,26,27,28]. Overall, the compounds investigated could be organized into four main medicinal-chemistry profiles. The most balanced and conventionally drug-like space was occupied by the leaf alkaloids O,O-dimethylcoclaurine, Nornanternine, (+)-Armepavine, and Corydine, together with Rosiglitazone. These compounds combined moderate molecular weight (313.17–357.11 Da), low-to-moderate TPSA (39.72–71.53 Å^2^), moderate lipophilicity (logP2.017–2.667), attractive QED values (0.821–0.941), and acceptance by Lipinski, Pfizer, GSK, and Golden Triangle filters, without PAINS alerts. Isouvaretin occupied a somewhat less favorable but still acceptable position, with intermediate QED (0.536) and full rule-based compliance, whereas Diuvaretin, despite Lipinski acceptance, already showed a weaker medicinal-chemistry profile because of its higher polarity (TPSA107.22 Å^2^), higher lipophilicity (logP4.629), rejection by the GSK rule, and poor QED (0.233). The oxadiazole reference ligand also remained within a relatively balanced space, with acceptable Lipinski and Golden Triangle compliance and the highest QED among the non-Rosiglitazone synthetic ligands (0.646), although it was rejected by the GSK rule.

A second profile corresponded to compounds that remained acceptable by some rule-based filters but were less optimal overall. This included 5-chloro-1-(4-chloroben-zyl)-3-(phenylthio)-1H-indole-2-carboxylic acid and 4-bromo-3-(carboxymethoxy)-5-{3-[(3,3,5,5-tetramethylcyclohexyl)amino]phenyl}thiophene-2-carboxylic acid, which combined acceptable or borderline Lipinski-related properties with lower QED values (0.378 and 0.402, respectively), increased lipophilicity, and rejection by the GSK rule for both ligands and by Golden Triangle for the brominated thiophene derivative. These profiles remain compatible with pharmacological utility but are less attractive from a classical oral medicinal-chemistry perspective.

A third profile comprised the highly lipophilic, high-molecular-weight, and highly flexible metabolites, represented mainly by the acetogenins Solamin, cis-Uvariamicin I, Uvariamicin II, Uvariamicin-I, Squamocin, and Annotemoyin-1. All exceeded 500 Da, showed logP values from 5.254 to 9.026, very low QED values (0.065–0.081), and rejection by Lipinski, GSK, and Golden Triangle criteria. Their high flexibility, reflected by 25–29 rotatable bonds and high flexibility indices, may support conformational adaptation within binding pockets but is generally unfavorable for oral developability and robust drug-likeness. Uvarinol also clustered closer to this less favorable space than to the balanced flavonoid profile, since it combined high molecular weight (574.20 Da), high TPSA (127.45 Å^2^), relatively high lipophilicity (logP5.772), low QED (0.146), and rejection by Lipinski, GSK, and Golden Triangle filters.

A fourth profile included the highly polar and strongly hydrophilic reference compounds α-acarbose, ADP-ribose, and α-cyclodextrin. These molecules displayed extremely high TPSA values (291.52–474.90 Å^2^), very low or negative logP values (−3.161 to −5.335), poor QED values (0.103–0.140), and poor compliance with classical developability filters, which is fully consistent with poor passive permeability and non-classical oral drug-like behavior. Their high Fsp3 or natural-product-like character does not compensate for their excessive size and polarity in the context of conventional small-molecule drug-likeness.

From a medicinal-chemistry perspective, the complete absence of PAINS alerts across the dataset supports the credibility of the predicted interactions and reduces the likelihood of nonspecific assay-interference behavior. Additional filters remained informative: several acetogenins, Uvarinol, α-acarbose, ADP-ribose, and α-cyclodextrin each showed one BMS alert, whereas Corydine and 5-chloro-1-(4-chloroben-zyl)-3-(phenylthio)-1H-indole-2-carboxylic acid each triggered one Chelator alert. Collectively, these data distinguish the most balanced candidates, especially O,O-dimethylcoclaurine, Nornanternine, (+)-Armepavine, Corydine, Rosiglitazone, and, to a lesser extent, Isouvaretin, from mechanistically active but weakly drug-like metabolites such as the acetogenins, Uvarinol, and the highly polar reference compounds.

## 3. Discussion

Diabetes remains a major global health burden, with its prevalence projected to reach 700 million cases by 2045 [29,30]. In this context, medicinal plants represent a valuable source of bioactive compounds capable of modulating multiple targets involved in glucose homeostasis [30]. Among them, *Uvaria chamae* has demonstrated antidiabetic potential in vitro and in vivo [16,17,18], which is further explored here using an integrated in silico approach combining docking, MM-GBSA, induced fit docking (IFD), QSAR, and ADMET analyses. While molecular docking provides an initial estimation of ligand–protein affinity [31,32], its limitations in predicting stable binding are well recognized, particularly for flexible ligands or large binding pockets [33,34]. The integration of MM-GBSA and IFD partially addresses these limitations by incorporating thermodynamic contributions and local receptor flexibility [35,36]. However, these approaches remain approximations and do not fully capture protein dynamics, which should be considered when interpreting residue-level interactions. In this study, compound prioritization was therefore based on the consistency across structural, QSAR (pIC_50_), and ADMET criteria rather than on isolated docking scores. Within this framework, stem- and root-derived flavonoids, particularly isouvaretin and diuvaretin, showed the most coherent profiles on PPARγ. These compounds combined favorable binding energies, stable induced-fit poses, and predicted activities close to reference ligands. Their interaction patterns involved residues such as Ser342 and Ser289, which is consistent with previous studies describing flavonoids as modulators of PPARγ through alternative binding regions distinct from classical full agonists [37,38,39]. Indeed, full agonists such as rosiglitazone typically stabilize the AF-2 region via interactions with Tyr473 and His449 [40,41,42], whereas flavonoids are more frequently associated with partial modulation mechanisms involving residues such as Ser342 [43,44,45]. The present observations are therefore consistent with reported structure–function relationships for this class of compounds. By contrast, acetogenins displayed strong MM-GBSA contributions but systematically deviated from drug-likeness criteria, including high molecular weight, extreme lipophilicity, low QED values, and predicted toxicity liabilities. This apparent discrepancy between structural scores and pharmacological relevance is consistent with known biases in structure-based methods, where large hydrophobic molecules can artificially benefit from favorable binding energies due to nonspecific interactions within hydrophobic pockets [44,46]. Similar observations have been reported in virtual screening studies of flexible natural products. In this context, acetogenins are more appropriately interpreted as structural probes or contributors to extract-level biological effects rather than as prioritized drug candidates. This interpretation aligns with the literature, where annonaceous acetogenins are mainly associated with mitochondrial or bioenergetic mechanisms rather than selective receptor modulation. The analysis of PTP1B further supports the relevance of flavonoids, with uvarinol showing the most consistent profile across structural and QSAR analyses. This is in agreement with numerous reports describing flavonoids such as quercetin, luteolin, and morin as PTP1B inhibitors with diverse inhibition mechanisms [47,48]. In contrast, evidence supporting acetogenins on this target remains limited and mostly indirect [49], which reinforces the cautious interpretation of their role in the present dataset. For digestive enzymes, the differences observed between porcine (3L2M) and human (1B2Y) α-amylase models can be explained by both structural and experimental factors. Despite high sequence similarity, small variations in the active site environment may affect ligand recognition [50,51]. In addition, the crystallographic structures correspond to different ligand-bound conformations, which is known to influence docking outcomes [52,53,54,55]. Such variability has been widely reported in structure-based studies and supports the use of multiple receptor conformations. In this context, the prioritization of the human model reflects its physiological relevance rather than a post hoc interpretation. Consistent with the literature, flavonoids remain well-established α-amylase inhibitors [51,56], while alkaloids show more heterogeneous activity profiles [51,57]. The DPP-4 analysis revealed a consistent contribution of leaf alkaloids, particularly nornanternine and (+)-armepavine, supported by both structural and QSAR data. This observation is consistent with the known role of DPP-4 inhibition in prolonging incretin hormone activity and improving glycemic control. However, these results remain predictive and should be interpreted as indicative of potential interactions rather than confirmed inhibitory mechanisms. SIRT6 interactions were mainly associated with acetogenins, which showed favorable structural profiles but limited pharmacological relevance due to their physicochemical and ADMET properties. Similar discrepancies between binding scores and developability have been reported for highly lipophilic natural products, reinforcing the need to interpret these results cautiously. Overall, the integration of structural, QSAR, and ADMET analyses supports a hierarchical interpretation in which flavonoids represent the most consistent and pharmacologically plausible candidates, alkaloids occupy an intermediate position, and acetogenins are deprioritized as drug-like leads despite favorable binding signals. This prioritization is based on the consistency of compounds across multiple criteria, including structural stability (docking, MM-GBSA, and induced-fit docking), predicted activity (QSAR pIC_50_), and developability (ADMET properties and drug-likeness). When considering the distribution of these compound classes across plant organs, a differentiated pattern emerges. Stem- and root-derived flavonoids are consistently associated with PPARγ modulation, while root-derived metabolites, including uvarinol and acetogenins, are more frequently linked to PTP1B and SIRT6 interaction profiles. In contrast, leaf-derived alkaloids are predominantly associated with DPP-4 inhibition and digestive enzyme targets such as α-amylase and maltase-glucoamylase. However, the role of acetogenins requires careful interpretation. Although these compounds are not prioritized as drug candidates due to their physicochemical and ADMET limitations, their recurrent structural contributions across multiple targets suggest that they may still participate in the overall biological activity of plant extracts, potentially through complementary or non-specific mechanisms. More broadly, the apparent organ-dependent distribution should be interpreted as a data-driven trend rather than a definitive biological specialization. Differences in phytochemical characterization across plant organs may introduce representation bias, and the observed associations may partly reflect compound availability rather than intrinsic organ-specific activity. Taken together, these findings provide a structured and transparent framework for prioritizing *Uvaria chamae* metabolites and their plant sources for future experimental investigation, while clearly distinguishing between pharmacologically plausible drug candidates and compounds contributing primarily to extract-level activity.

Limitations and translational implications of organ-based multitarget computational profiling

This organ-based hierarchy should be interpreted with considerable caution, as it arises from an inherently explorative in silico framework. The predictive strength of the analysis is constrained by structural limitations, including the uneven availability of high-resolution crystallographic data across molecular targets, which may introduce biases in docking accuracy and scoring reliability [34,58]. The reliance on predominantly rigid receptor models further limits the ability to capture conformational flexibility, induced-fit effects, solvent dynamics, and entropic contributions that are essential for realistic ligand–protein recognition under physiological conditions [59,60]. The main limitations of the QSAR models arise from the structural heterogeneity of the dataset, which may affect the robustness of predictions for certain chemical classes. In particular, compounds with extreme physicochemical properties may not be fully represented in the training space, limiting the reliability of their predicted activities. The integration of ADMET predictions must also be interpreted within their probabilistic nature, as these models provide estimations rather than experimentally validated pharmacokinetic or toxicological outcomes [61]. Consequently, their use in multi-parameter prioritization should be regarded as hypothesis-generating rather than confirmatory evidence.

Apparent organ-specific contributions may also reflect differences in the phytochemical characterization of *Uvaria chamae* across plant organs in the literature, potentially introducing representation bias in compound availability and downstream target prediction. In addition, the biological activity of crude extracts is likely governed by complex synergistic, additive, and antagonistic interactions among metabolites, which cannot be captured through single-compound docking approaches [62].

Overall, these findings support a multitarget pharmacological framework for *Uvaria chamae* P. Beauv., involving coordinated modulation of insulin sensitivity, metabolic regulation, incretin signaling, and postprandial glucose control. This model remains hypothesis-driven and requires experimental validation through enzymatic assays, cellular systems, and in vivo studies, complemented by molecular dynamics and free-energy calculations to improve mechanistic resolution.

## 4. Methods

### 4.1. Computational Resources

All computational analyses were performed on a Dell Pro 16 plus PB16250 laptop (Dell, Round Rock, TX, USA) equipped with an Intel(R) Core(TM) Ultra 7 255U processor (2.00 GHz) (Intel Corporation, Santa Clara, CA, USA), 16.0 GB of RAM, and a Windows 11 Education 64-bit operating system. The software tools used included Maestro V14.3. Additional analyses were performed using the Admetlab3 web platform (https://admetlab3.scbdd.com (accessed on 10 February 2026)). Protein structures were extracted from the RCSB Protein Data Bank (PDB), and phytochemical structures were obtained from PubChem (https://pubchem.ncbi.nlm.nih.gov/ (accessed on 3 December 2025)) and Coconut Natural Products (https://coconut.naturalproducts.net/ (accessed on 3 December 2025)).

### 4.2. Protein Selection for Docking

Biological assemblies of enzymes known to play a crucial role in diabetes were selected for in silico studies with PDB IDs: 2Q5S [45], 3L2M [52], 2PRG [43], 3C45 [63], and 2QBQ [64], 3K35 [65], 2QMJ [66], 1B2Y [53]. The 3D structures of these proteins and their respective PDB IDs are shown in Table 4. All the proteins mentioned were extracted from the site https://www.rcsb.org (accessed on 6 December 2025) and processed by the Maestro Schrödinger protein pretreatment module version 14.3.

### 4.3. Protein Preparation for Docking

The proteins used for molecular docking were prepared using the Protein Preparation Assistant [68]. This preparation involved the removal of heteroatoms and crystallographic water molecules, except for essential structural water molecules. Missing side chains were fixed, and hydrogen atoms were added according to physiological pH. The protonation states of the titratable residues were adjusted using PROPKA at pH7.4. After optimization and minimization of the protein structures, a docking grid was generated with the Glide application around the active site defined by the co-crystallized ligand; the grid center coordinates for each receptor are shown in Table S1, with a grid size set to 20 Å. Molecular docking was performed using the Glide module on all compounds, which were docked sequentially in Standard Precision (SP) and Extra Precision (XP) modes. Subsequently, all resulting ligand-protein complexes were subjected to post-docking refinement using the Prime MM-GBSA approach to estimate the binding free energies (Δ G_bind).

### 4.4. Selection and Preparation of Ligands

The three-dimensional structures of compounds from *Uvaria chamae* P. Beauv were identified through a literature review of the PubMed, Scopus, ScienceDirect, and Google Scholar databases. The search was conducted using keywords such as “*Uvaria chamae*,” “phytochemistry,” “phytochemical constituents,” “secondary metabolites,” and “chemical composition.” Studies were included if they reported the isolation or identification of chemical compounds from different parts of the plant (seeds, leaves, stems, and Root). Articles describing clearly characterized chemical structures were retained, while publications not reporting compounds specific to *Uvaria chamae* P. Beauv, redundant data, or studies without reliable structural identification were excluded. A total of 106 molecules were identified from different parts of the plant. However, only 105 compounds were retained for the in silico analyses, as the structure of one reported compound, joolanin, could not be reliably assigned from the available literature due to ambiguity regarding a possible alternative inverse form. The list of retained compounds, together with their botanical origins and chemical families, is presented in Table 5.

The ligands were then prepared using LigPrep (Maestro version 14.3, Schrödinger, LLC, New York, NY, USA), including energy minimization, evaluation of possible ionization states, and parameterization with the OPLS4 force field [69]. The standard molecules were prepared using the same procedure for redocking, based on their crystallographic conformations.

**Table 5:** *Uvaria chamae* P. Beauv compounds selected for in silico study.

N°	Name	PubChem/Coconut ID	Molecular Formula	Part of the Plant	References
**Acetogenins**					
1	Annotemoyin-1	73029717	C_35_H_64_O_5_	Root	[70]
2	Bullatencin	44577080	C_37_H_66_O_5_	Root	[70,71]
3	Chamuvarinin	11342455	C_37_ H_64_ O_6_	Root, seeds	[72,73,74]
4	Cis-bullatencin	CNP0239276.2	C_37_ H_66_ O_5_	Root	[70]
5	Cis-uvariamicin-I	14759336	C_37_ H_68_ W_5_	Root	[70]
6	Desacetyluvaricin	127149	C_37_ H_66_ W_6_	Root, seeds	[73,74]
7	Dieporeticanin 1	102064358	C_37_ H_66_ W_4_	Seeds	[73]
8	Dieporeticanin 2	102064359	C_37_ H_66_ W_4_	Seeds	[73]
9	Dieporeticenin	101420981	C_37_H_64_ W_4_	Seeds	[73]
10	Joolanin	-	C_37_ H_64_ O_7_	Seeds	[73]
11	Neoannonin	76315048	C_35_ H_62_ O_6_	Root	[74]
12	Reticulatacin	10438442	C_37_ H_68_ O_5_	Root	[70]
13	Solamin	11376469	C_35_ H_64_ O_5_	Root	[70]
14	Squamocin	441612	C_37_ H_66_ O_7_	Root, seeds	[73,74]
15	Tripoxyrollin	131753021	C_37_ H_64_ O_5_	Seeds	[73]
16	Uvariamicin-I	44577078	C_37_ H_68_ O_5_	Root	[70]
17	Uvariamicin-II	101392149	C_38_ H_70_ O_5_	Root	[70]
18	Uvariamicin-III	44577079	C_37_ H_68_ O_5_	Root	[70]
**Alkaloids**					
19	(+)-Armepavine	680292	C_19_H_23_NO_3_	Leaves	[75]
20	Corydine	CNP0157012.2	C_20_ H_23_ NO_4_	Leaves	[75]
21	Nantenine	197001/CNP0165224.1	C_20_ H_21_ NO_4_	Leaves	[75]
22	Nornantenine	3084228	C_19_ H_19_ NO_4_	Leaves	[75]
23	O,O-Dimethylcoclaurine	10829011	C_19_ H_23_ NO_3_	Leaves	[75]
**Flavonoids**					
24	Chametin/Chamanetin	21721821	C_22_ H_18_ O_5_	Stem, Root	[71,76,77]
25	Chamuvaritin	100418	C_29_ H_24_ O_5_	Root	[78,79]
26	Dichamanetin	181193	C_29_ H_24_ O_6_	Stem, Root	[71,76,77]
27	Diuvaretin	3085222	C_30_H_28_O_6_	Stem, Root	[71,77,80]
28	Isochamanetin	5318528	C_22_ H_18_ O_5_	Stem, Root	[71,77]
29	Isouvaretin	151670	C_23_ H_22_ O_5_	Stem, Root	[71,76,77]
30	Pinocembrin	68071	C_15_H_12_O_4_	Stem	[77]
31	Pinostrobin	73201	C_16_H_14_O_4_	Root	[77]
32	Uvangoletin	6483649	C_16_H_16_O_4_	Root	[11]
33	Uvaretin	73447	C_23_ H_22_ O_5_	Stem, Root	[76,79]
34	Uvarinol	21721823	C_36_ H_30_ O_7_	Stem, root	[71,81]
**Phenols**					
35	Caffeic acid	689043	C_9_H_8_O_4_	Root	[82]
36	Ellagic acid	5281855	C_14_H_6_O_8_	Root	[82]
37	Proanthocyanidin	108065	C_31_ H_28_ W_12_	Root	[82]
38	Resorcinol	5054	C_6_H_6_O_2_	Root	[82]
**Essential oils, sterols and terpenes**					
39	Beta-sitosterol	222284	C_29_ H_50_ W	Stem	[83]
40	Stigmasterol	5280794	C_29_ H_48_ W	Stem	[83]
41	1-epi-cubebol	91753170	C_15_ H_26_ O	Leaves, Root	[19,72]
42	1-nitro-2-phénylethane	80208	C_8_H_9_NO_2_	Leaves	[19]
43	3-Carene	26049	C_10_ H_16_	Leaves	[19]
44	3Z-Hexenylbenzoate	101687121	C_13_ H_16_ O_2_	Leaves	[19]
45	4-nitrophenyl laurate	74778	C_18_ H_27_ N W_4_	Stem	[83]
46	6,9-Guaiadiene	527113	C_15_ H_24_	Leaves	[19]
47	Alloaromadendrene	10899740	C_15_ H_24_	Leaves	[19]
48	Alpha-cadinol	10398656	C_15_H_26_O	Leaves	[19]
49	Alpha-copaene	19725	C_15_ H_24_	Leaves	[19,72]
50	Alpha-cubebene	442359	C_15_ H_24_	Leaves	[19]
51	Alpha-farnesene	5281516	C_15_ H_24_	Leaves	[19]
52	Alpha-Muurolene	12306047	C_15_ H_24_	Leaves	[19]
53	Alpha-phellandrene	7460	C_10_ H_16_	Leaves	[19]
54	Alpha-santalene	94164		Stem	[83]
55	Alpha-Santalone	162952798	C_15_H_22_O	Leaves	[19]
56	Alpha-terpinene	7462	C_10_ H_16_	Leaves	[19]
57	Alpha-terpineol	17100	C_10_H_18_O	Leaves	[19]
58	Baldrinal	159846	C_12_H_10_O_4_	Stem	[83]
59	benzaldehyde	240	C_7_H_6_O	Leaves	[19]
60	Benzeneacetonitrile	8794	C_8_ H_7_ N	Leaves	[19]
61	Benzyle benzoate	2345	C_14_H_12_O_2_	Leaves	[19]
62	beta-bourbonene	62566	C_15_ H_24_	Leaves	[19]
63	beta-copaene	57339298	C_15_ H_24_	Leaves	[19]
54	beta-cubebene	93081	C_15_ H_24_	Leaves	[19]
65	Beta-elemene	6918391	C_15_ H_24_	Leaves	[19]
66	beta-Maaliene	101596917	C_15_ H_24_	Stem	[83]
67	beta-ocimene	18756	C_10_ H_16_	Leaves	[19]
68	beta-selinene	442393	C_15_ H_24_	Leaves	[19]
69	Bicyclogermacrene	13894537	C_15_ H_24_	Leaves	[19]
70	Borneol	64685	C_10_H_18_O	Leaves	[19]
71	Camphene	6616	C_10_ H_16_	Leaves	[19]
72	Caryophyllene	5281515	C_15_ H_24_	Leaves	[19]
73	Caryophyllene oxyde	1742210	C_15_H_24_O	Leaves	[19]
74	Copaene	12303902	C_15_ H_24_	Leaves	[19]
75	Cubebol	11276107	C_15_H_26_O	Leaves	[19]
76	delta-cadinene	441005	C_15_ H_24_	Leaves	[19]
77	Elemol	92138	C_15_H_26_O	Leaves	[19]
78	E-phytol	5280435	C_20_H_40_O	Leaves	[19]
79	Epi-cubebol	91753433	C_15_H_24_O	Leaves	[19]
80	Epicubenol	12046149	C_15_H_26_O	Leaves	[19]
81	Eucalyptol	2758	C_10_H_18_O	Leaves, Root	[19,72]
82	gamma-Muurolene	12313020	C_15_ H_24_	Leaves	[19]
83	gamma-terpinene	7461	C_10_ H_16_	Leaves	[19]
84	Germacrene A	9548705	C_15_ H_24_	Leaves	[19]
85	Germacrene B	5281519	C_15_ H_24_	Leaves	[19]
86	Germacrene D	5317570	C_15_ H_24_	Leaves, Root	[19]
87	Gleenol	6429080	C_15_H_26_O	Leaves	[19]
88	Humulene	5281520	C_15_ H_24_	Leaves, Root	[19]
89	Humulene epoxide II	10704181	C_15_H_24_O	Leaves	[19]
90	Isopropyle linoleate	5352860	C_21_ H_38_ O_2_	Stem	[83]
91	Limonene	22311	C_10_ H_16_	Leaves	[19]
92	Linalol	6549	C_10_H_18_O	Leaves, Root	[19,72]
93	Lupeol	259846	C_30_H_50_O	Stem	[83]
94	Myrcene	31253	C_10_ H_16_	Leaves	[19]
95	Nerolidol	5284507	C_15_H_26_O	Leaves	[19]
96	P-cymene	7463	C_10_H_14_	Leaves	[19]
97	Pentadecanal	17697	C_15_H_3_O	Leaves	[19]
98	Spathulenol	92231	C_15_H_24_O	Leaves	[19]
99	Squalene	638072	C_30_H_50_	Stem	[83]
100	tau-Cadinol	160799	C_15_H_26_O	Leaves	[19]
101	tau-Muurolol	6432221	C_15_H_26_O	Leaves	[19]
102	Terpinen-4-ol	11230	C_10_H_18_O	Leaves	[19]
103	trans-Cadina-1(6),4-diene	10798255	C_15_H_24_	Leaves	[19]
104	trans-Cadina-1,4-diene	6430869	C_15_H_24_	Leaves	[19]
105	trans-Muurola-4(14),5-diene	91747125	C_15_H_24_	Leaves	[19]
**Fatty acids**					
106	Palmitic acid	985	C_16_H_32_O_2_	Leaves	[19]

### 4.5. Molecular Docking Procedure

Molecular docking simulations were performed using the Glide module of the Schrödinger suite (version 2025-1) in extra precision (XP) mode to evaluate the binding affinity of ligands to the active sites of target proteins [84]. The ligands were docked into the corresponding binding pockets and ranked according to their Glide XP docking scores. Docking reliability was assessed by redocking selected co-crystallized ligands, which yielded satisfactory RMSD (RMSD should be <2Å) values for 2Q5S, 2PRG, 2QBQ, 3K35, 3C45, and 2QMJ (0.39, 1.18, 0.53, 0.98, 1.03, and 1.53 Å, respectively). To reduce potential selection bias, all docked ligands were subsequently evaluated by Prime MM-GBSA using the VSGB2.0 implicit solvation model and the OPLS4 force field, thereby providing a complementary assessment of the relative stability of the predicted protein–ligand complexes. Thereafter, the complexes showing the most relevant binding profiles were subjected to induced fit docking (IFD) to account for local active-site flexibility and to further refine ligand–protein binding conformations.

### 4.6. In Silico Evaluation of Biological Activity

A quantitative structure–activity relationship (QSAR) analysis was performed to estimate the predicted biological activity of the compounds as pIC_50_ values [85]. Due to the limited availability of experimental data for some targets, QSAR models could only be developed for proteins with a sufficient number of inhibitors reported in the literature. Thus, QSAR modeling was conducted for PPARγ (2Q5S, 2PRG), PTP1B (2QBQ), maltase-glucoamylase (2QMJ), and DPP-4 (3C45). The other targets described by docking were not included in the QSAR modeling due to an insufficient amount of activity data available for model training.

Data sets of experimentally validated inhibitors, along with their biological activities expressed as pIC_50_, were retrieved from the ChEMBL database (https://www.ebi.ac.uk/chembl/ (accessed on 5 January 2026) using the corresponding protein targets. Molecular descriptors were generated using the AutoQSAR module implemented in Schrödinger Maestro. These descriptors include a broad range of physicochemical, topological, and structural properties relevant for ligand–target interactions.

For each protein target, independent QSAR models were constructed using the corresponding dataset. The data were randomly divided into a training set (75%) and an external test set (25%) to enable model construction and validation. QSAR models were developed to establish quantitative relationships between molecular descriptors and biological activity (pIC_50_ values) [83]. The AutoQSAR workflow automatically selects the most relevant descriptors and builds predictive models using multiple machine learning algorithms, including kernel-based partial least squares (KPLS), radial, dendritic, and linear models, as reported in Table 6.

Model performance was evaluated using standard statistical parameters, including the coefficient of determination for the training set (R^2^(train)), the predictive coefficient obtained from the AutoQSAR workflow (Q^2^), and the root mean square error (RMSE). In addition, the external predictive performance of each model was explicitly assessed by calculating the coefficient of determination on the independent test set (R^2^(test)) using the observed and predicted pIC_50_ values.

The developed models exhibited R^2^(train) values ranging from 0.66 to 0.75, while the manually calculated R^2^(test) values ranged from 0.67 to 0.74, indicating good predictive performance and generalization ability. The Q^2^ values (>0.60) were consistent with the external validation results and further supported the robustness of the models. RMSE values (0.48–0.72) confirmed an acceptable level of prediction error in pIC_50_ units. The close agreement between R^2^(train), Q^2^, and R^2^(test) suggests the absence of significant overfitting.

The applicability domain (AD) of the models was considered qualitatively in terms of chemical space coverage. Due to the structural diversity of the dataset, particularly the presence of large and flexible compounds such as acetogenins, some predictions may involve extrapolation beyond the training domain and should therefore be interpreted with caution. The descriptor selection process is model-dependent and varies according to each protein target and algorithm, allowing the identification of target-specific structure–activity relationships. Descriptor contributions were evaluated using the built-in feature importance analysis within the AutoQSAR framework.

Overall, the QSAR models demonstrated consistent predictive performance across multiple targets and provided complementary insights to molecular docking analyses, supporting the prioritization of bioactive compounds.

### 4.7. Drug-Likeness Assessment and ADMET Property Prediction

To evaluate the drug-like potential and ADMET characteristics of the selected molecules, analyses were performed using the ADMETLab 3.0 web server [84,85]. This platform uses a deep multitasking neural network (DMPNN) model, trained on a large dataset of ADMET endpoints, to simultaneously predict pharmacokinetic properties (absorption, distribution, metabolism, and excretion), physicochemical parameters, and medicinal chemistry descriptors. For this purpose, SMILES representations of the studied ligands were imported into the software, which then generated predictions regarding drug-like potential, pharmacodynamic properties, and pharmacokinetic profiles [23,84].

## 5. Conclusions

In conclusion, this study highlights a polypharmacological mixture-based antidiabetic potential of *Uvaria chamae*, with distinct contributions from different plant organs. Stem- and root-derived flavonoids, particularly isouvaretin and diuvaretin, together with uvarinol, emerged as the most consistent candidates based on the integration of structural, QSAR, and ADMET analyses, showing favorable predicted activity and acceptable pharmacokinetic and toxicity profiles. Leaf alkaloids, including (+)-armepavine, nornanternine, and corydine, displayed complementary profiles, particularly in DPP-4 and digestive enzyme inhibition, with overall favorable drug-likeness. In contrast, acetogenins, despite favorable binding energies, were not prioritized as drug-like leads due to their high lipophilicity, low QED values, and predicted toxicity liabilities, although they may contribute to extract-level biological activity. These findings provide testable hypotheses for experimental validation through enzymatic assays, cell-based models of glucose metabolism, and gene expression analyses of key metabolic regulators, and offer a structured framework for the rational prioritization of *Uvaria chamae* metabolites.

## Figures and Tables

**Table 1:** Analysis of the molecular interactions of the compounds.

Protein ID	ID	Molecule Name	Organs	Family	Binding Energy (kcal/mol)	MMGBSA dG (kcal/mol)	Number of Hydrogen Bond Formed	Residues Involved in Hydrogen Bond Formation	Residues Involved in Hydrophobic Interaction	Residues Involved in Polar Interaction	Residues Involved in π–π Stacking
2Q5S	151670	Isouvaretin	Stem/Root	Flavonoid	−10.75	−75.46	1	SER342	LEU333-LEU330-ALA292-ILE326-VAL339-LEU340-ILE341-PHE264-ILE262-ILE281-LEU255-ILE249-CYS285-MET348	-	-
	3085222	Diuvaretin	Stem/Root	Flavonoid	−9.24	−69.62	3	SER342-LEU340-ARG288	LEU228-PRO227-PHE226-ILE296-ALA292-LEU333-LEU330-MET329-ILE326-CYS285-MET348-MET364-LEU353-ILE281-VAL339-LEU340-ILE341	-	-
	NZA	5-chloro-1-(4-chlorobenzyl)-3-(phenylthio)-1H-indole-2-carboxylic acid	Standard	-	−10.80	−71.01	1	SER342	LEU333-LEU330-ALA292-ILE326-VAL339-LEU340-ILE341-MET364-PHE363-LEU353-CYS285-ILE249-MET348-LEU255-ILE281-ILE262-PHE264	-	-
2PRG	11376469	Solamin	Root	Acetogenins	−9.10	−79.73	2	SER342-GLU291	ILE249-PHE247-LEU270-MET348-ILE281-ILE262-CYS285-PHE287-ILE341-LEU353-LEU340-VAL339-LEU333-MET364-PHE363-LEU330-TYR327-ILE326-LEU469-TYR473	-	-
	151670	Isouvaretin	Stem/Root	Flavonoid	−9.71	−68.88	1	LEU340	LEU340-ILE341-VAL339-LEU333-LEU330-ALA292-TYR327-ILE326-TYR473-LEU469-CYS285-PHE282-MET364-PHE363	HIE449	-
	10829011	O,O-Dimethylcoclaurine	Leaves	Alkaloid	−6.69	−64.68	3	HIE449-HIE323-ARG288	PHE282-LEU453-PHE363-MET364-ILE326-TYR327-MET329-LEU330-LEU333-ALA292-CYS285-LEU465-LEU469-TYR473	-	-
	445655	Rosiglitazone	Standard	-	−12.06	−65.84	3	TYR473-SER289-GLN286	MET348-LEU353- LEU333-LEU330-MET364-TYR327-PHE363-ILE326-LEU-469-TYR473 -CYS285-VAL339-LEU340- ILE341-ILE281-LEU465	-	-
2QBQ	14759336	Cis-Uvariamicin	Root	Acetogenins	−2.991	−71.95	2	LYS120-GLY259	MET258-ALA27-TYR20-ALA264-ALA17-PHE182-TYR46-VAL49-ILE219-ALA217	-	-
	21721823	Uvarinol	Stem/Root	Flavonoid	−5.222	−63.62	1	TYR46	TYR46-VAL49-LEU119-LEU88-CYS215-ALA217-ILE219-PHE182	-	-ARG45 (pi-cation) -PHE182 (pi-pi stacking)
	441612	Squamocin	Root/Seeds	Acetogenins	−3.061	−62.69	3	GLY259-GLN262-LYS120	VAL49-TYR46-ILE219-ALA217-PHE182-MET258-ALA27	-	-
	17759043	4-Bromo-3-(Carboxymethoxy)-5-{3-[(3,3,5,5-Tetramethylcyclohexyl)amino]phenyl}thiophene-2-Carboxylic Acid	Standard	-	−9.28	−72.96	6	GLN262-LYS120-ARG221-CYS215-PHE182-GLN266	MET258-VAL49-TYR46-ILE219-ALA217-PHE182-CYS215	-GLY220 (Halogen bond) -LYS120-ARG221 (Salt bridge)	-
3K35	14759336	cis-Uvariamicin I	Root	Acetogenins	−1.409	−84.63	4	HIS131-GLN111-LYS13	ALA51-PHE62-LEU239-TYR255-PRO65-TRP69-ILE183-LEU184-TRP186-LEU190-PRO219-ILE217-VAL113-LEU215	-	-
	101392149	Uvariamicin II	Root	Acetogenins	−0.259	−78.59	2	ALA51-GLN111	TRP69-ILE217-LEU215-LEU239-PRO65-TYR255-PHE62-PRO60-ALA51-MET155-ILE183-LEU184-TRP186-VAL113	-	-
	44577078	Uvariamicin-I	Root	Acetogenins	−1.702	−73.34	1	ALA51	LEU190-TRP186-PRO219-ILE217-LEU184-TRP69-LEU215-PHE62-ALA51-PRO65-LEU239-PHE22-TYR255-VAL256	-	-
	445794	Adp-ribose	Standard	-	−15.95	−99.88	14	VAL256-THR55-ASN238-LEU239-GLN240-HIS131-ARG63-SER214-THR213-PHE62-ALA51	TYR255-VAL256-ALA56-VAL237-LEU239-VAL113-TRP186-TRP69-ILE217-PRO65-LEU215-PHE62-ALA51		-
3L2M	101392149	Uvariamicin II	Roots	Acetogenins	−4.68	−62.09	2	ASP356-SER55	LEU165-VAL163-TRP59-TRP357-VAL354-ALA107-ALA108-ILE49-VAL50-VAL51-PRO54	-	-
	3085222	Diuvaretin	Stem/Root	Flavonoid	−6.09	−57.53	2	VAL163-GLN63	LEU165-VAL163-CYS103-TRP59-TRP357-VAL354-ALA107-ALA108-ILE49-VAL50-VAL51-PRO54	-	-
	CNP0240351.2	Annotemoyin-1	Root	Acetogenins	−4.02	−56.44	3	ASP356-THR52	VAL354-TRP357-PRO54-VAL51-VAL50-ILE49-ALA107-ALA108-LEU165-VAL163-TRP59	-	-
	444913	Alpha cyclodexrine	Standard	-	−7.87	−34.61	3	ASN53-ALA108	PRO54-TRP59-ALA107-ALA108	-	-
1B2Y	680292	(+)-Armepavine	Leaves	Alkaloid	−5.66	−47.21	2	TYR151-ASP300	LEU162-ILE235-ALA198-TYR62-TRP59-TRP58-TRP434-TYR151-TYR276-MET274	-GLU233, ASP300 (Salt bridge)	- TYR62 (Pi cation)
	445421	Alpha ascarbose	Standard	-	−13.43	−48.72	6	TYR151-THR163- HIS201-GLU233-ASP197	LEU165-LEU162-TYR151-ALA198-ILE235-VAL98-TYR62-TRP59-TRP58	-	-
2QMJ	CNP0157012.2	Corydine	Leaves	Alkaloid	−3.60	−46.38	0	-	PHE450-MET444-TRP406-TYR299-TYR605-PHE575-ALA576-LEU577	-ASP542 (Salt bridge)	PHE575 (pi-pi stacking)
	445421	Alpha ascarbose	Standard	-	−10.27	−29.02	7	ASP443-ASH327-HIE600-ASP203-ASP542-THR205	TRP406-TYR299-TRP539-MET444-ILE328-TRP441-ILE364-PHE575-ALA576-LEU577-TYR605-PHE450	-	-
3C45	3084228	Nornanternine	Leaves	Alkaloid	−8.33	−62.06	3	TYR547-GLU205-GLU206	VAL711-TYR662-TRP659-TYR631-VAL656-TYR666-TYR547-PRO550-CYS551-PHE357-TYR670	GLU206-GLU205 (Salt bridge)	-
	680292	(+)-Armepavine	Leaves	Alkaloid	−6.03	−55.18	4	TYR666-PRO550-GLN553-TYR585	TYR666-PRO550- TYR585-CYS551-TYR670-TYR547-PHE347-TYR456	-	PHE347 (Pi catin, Pi stacking)
	24768547	(2S,3S)-3-{3-[2-chloro-4-(methylsulfonyl)phenyl]-1,2,4-oxadiazol-5-yl}-1-cyclopentylidene-4-cyclopropyl-1-fluorobutan-2-amine	Standard	-	−9.17	−58.03	3	TYR662-GLU206-GLU205	TYR631-VAL711-TYR662-TRP659-VAL656-PHE357-TYR547-CYS551-TYR666-PRO550	-GLU205, GLU206 (Salt bridge) -PRO550 (Halogen bond)	TYR547-PHE357 (Pi Stacking)

**Table 2:** Structural validation by Induced Fit Docking of major ligands selected from XP docking and MM-GBSA analyses.

Protein ID	ID	Molecule Name	Organ	Family	Initial Binding Energy (kcal/mol)	IFD Binding Energy (kcal/mol)	ΔG_IFD_	Number of Hydrogen Bond Formed	Residues Involved in Hydrogen Bond Formation
2PRG	11376469	Solamin	Root	Acetogenins	−9.10	-	-	-	-
	151670	Isouvaretin	Stem/Root	Flavonoid	−9.71	−11.545	−68.88	1	SER289
	10829011	O,O-Diméthylcoclaurine	Leaves	Alkaloid	−6.69	−7.545	−64.68	2	HIE449-HIE323
	445655	Rosiglitazone	Standard	-	−12.06	−11.501	−65.87	4	HIE449-GLN286-SER289-HIE323
2Q5S	151670	Isouvaretin	Stem/Root	Flavonoid	−10.75	−13,100	−72.45	2	GLU25- SER342
	3085222	Diuvaretin	Stem/Root	Flavonoid	−9.24	−12.69	−49.11	3	SER34-GLU343-ILE281
	NZA	5-chloro-1-(4-chlorobenzyl)-3-(phenylthio)-1Hindole-2-carboxylic acid	Standard	-	−10.80	−12.70	−71.01	2	SER34-ARG288
2QBQ	14759336	Cis-Uvariamicin	Root	Acetogenins	−2.991	−5.87	−71.95	2	SER216-LYS36
	21721823	Uvarinol	Stem/Root	Flavonoid	−5.222	−8.39	−63.62	4	ASP48-ASP29-ARG24-ARG254
	441612	Squamocin	Root/Seeds	Acetogenins	−3.061	−5	−62.69	4	ARG24-LYS120-ARG221
	17759043	4-Bromo-3-(Carboxymethoxy)-5-{3-[(3,3,5,5-Tetramethylcyclohexyl)amino]phenyl}thiophene-2-Carboxylic Acid	Standard	-	−9.289	−8.55	−72.96	5	ARG221-PHE182-GLN266-GLN262
3K35	14759336	cis-Uvariamicin I	Root	Acetogenins	−1.409	−9.02	−84.63	3	ASP81-SER214-ARG63
	101392149	Uvariamicin II	Root	Acetogenins	−0.259	−6.22	−78.59	1	GLU20
	44577078	Uvariamicin-I	Root	Acetogenins	−1.702	−7.55	−73.34	3	LEU184-GLN240-LEU239
	445794	Adp-ribose	Standard	-	−16.208	−17.36	−99.88	12	HIS131-SER214-THR213-GLN240-LEU239- -ASN238-VAL256-ARG63-PHE62-ASN112-ASN238-GLN111
1B2Y	680292	(+)-Armepavine	Leaves	Alkaloid	−5.66	−7.35	−47.21	3	ASP300-TYR151-HIS201
	445421	Alpha acarbose	Standard	-	−13.56	−17.42	−48.72	9	ASP300-THR163-GLU233-GLU240-GLY306-HIE305-GLN63
3L2M	101392149	Uvariamicin II	Root	Acetogenins	−4.68	−8.155	−62.09	3	ASP300-GLN63-ASP197
	3085222	Diuvaretin	Stem/root	Flavonoid	−6.09	−12.109	−57.53	3	THR213-GLN111-ASP61
	CNP0240351.2	Annotated average-1	Root	Acetogenins	−4.02	−5.79	−56.44	1	SER214
	444913	Alpha cyclodexrine	Standard	-	-	-	-	-	-
2QMJ	CNP0157012.2	Corydine	Leaves	Alkaloid	−3.60	−7.84	−46.38	2	ASP542-TRP406
	445421	Alpha acarbose	Standard	-	−13.70	−12.32	−29.02	11	THR205-ASP203-ASP443-ASH327-HIE600-ASP542-ARG526-LYS480
3C45	3084228	Nornanternine	Leaves	Alkaloid	−8.33	−9.97	−62.07	1	GLU205
	680292	(+)-Armepavine	Leaves	Alkaloid	−6.03	−9.701	−55.18	2	SER630-TYR547
	24768547	(2S,3S)-3-{3-[2-chloro-4-(methylsulfonyl)phenyl]-1,2,4-oxadiazol-5-yl}-1-cyclopentylidene-4-cyclopropyl-1-fluorobutan-2-amine	Standard	-	−9.17	−11.438	−58.03	4	GLU206-GLU205-TYR662-GLN553

**Table 3:** QSAR predicted pIC_50_ values for selected *Uvaria chamae* P. Beauv compounds on protein targets involved in glucose homeostasis.

Protein ID	Molecule Name	Organ	Family	pIC_50_ (Predicted)
PPARγ (2Q5S)	Isouvaretin	Stem/Root	Flavonoid	6.224
	Diuvaretin	Stem/Root	Flavonoid	6.204
	Solamin	Root	Acetogenins	5.009
	Isouvarin	Stem/Root	Flavonoid	6.224
	O,O-Dimethylcoclaurine	Leaves	Alkaloid	6.169
	5-chloro-1-(4-chlorobenzyl)-3-(phenylthio)-1H-indole-2-carboxylic acid	Standard	-	6.317
	Rosiglitazone	Standard	-	6.417
PTP1B (2QBQ)	Cis-Uvariamicine	Root	Acetogenins	5.556
	Uvarinol	Stem/Root	Flavonoid	5.426
	Squamocin	Root/Seeds	Acetogenins	5.556
	4-Bromo-3-(Carboxymethoxy)-5-{3-[(3,3,5,5-Tetramethylcyclohexyl)amino]phenyl}thiophene-2-Carboxylic Acid	Standard	-	5.219
Maltase-glucoamylase (2QMJ)	Corydine	Leaves	Alkaloid	4.960
	Alpha acarbose	Standard	-	4.123
DPP-4 (3C45)	(+)-Armepavine	Leaves	Alkaloid	6.557
	Nornanternine	Leaves	Alkaloid	5.925
	(2S,3S)-3-{3-[2-chloro-4-(methylsulfonyl)phenyl]-1,2,4-oxadiazol-5-yl}-1-cyclopentylidene-4-cyclopropyl-1-fluorobutan-2-amine	Standard	-	7.100

**Table 4:** Target proteins selected for the in silico study of antidiabetic potential.

Protein	Function	ID PDB	Reference
PPARGAMMA linked to a partial agonist NTZDPA	Controls glucose and lipid metabolism	2Q5S	[45]
Porcine pancreatic alpha-amylase with alpha-cyclodextrin	Responsible for the absorption of glucose into the blood	3L2M	[52]
Structure of human pancreatic alpha-amylase in complex with the carbohydrate inhibitor, acarbose	Responsible for the absorption of glucose into the blood	1B2Y	[53]
Human peroxisome proliferator-activated receptor gamma ligand-binding domain	Ligand-dependent transcription factor essential for adipocyte differentiation and glucose homeostasis.	2PRG	[67]
Protein tyrosine phosphatase 1B	Negative regulator of insulin and leptin receptor signaling pathways	2QBQ	[64]
Human dipeptidyl peptidase IV/CD26 in complex with a fluoro-olefin inhibitor	Deactivates the natural hypoglycemic incretin hormone GLP-1, restoring glucose homeostasis	3C45	[63]
Crystal Structure of Human SIRT6	SIRT6 helps lower blood sugar levels	3K35	[65]
N-terminal human maltase-glucoamylase crystalline complex with acarbose	Responsible for the hydrolysis of starch end products into glucose	2QMJ	[66]

**Table 6:** Datasets and statistical performance of the QSAR models developed for the studied targets.

ID PDB	Total Number of Inhibitors	Training Set (75%)	Test Set (25%)	R^2^ (Train)	Q^2^	R^2^ (Test)	RMSE (Test)	SD (Train)	Ranking Score	Model
**2Q5S**	525	394	131	0.75	0.70	0.74	0.64	0.59	0.66	kpls_radial_3
**2QBQ**	448	336	112	0.67	0.68	0.67	0.54	0.54	0.66	kpls_radial_12
**2QMJ**	304	228	76	0.66	0.67	0.67	0.48	0.50	0.67	kpls_dendritic_12
**3C45**	448	336	112	0.74	0.60	0.71	0.72	0.59	0.53	kpls_linear_1

## Data Availability

Data included in article/Supplementary Materials/referenced in article.

## Supplementary Materials

The following supporting information can be downloaded at: https://www.mdpi.com/article/10.3390/molecules31111879/s1, Figures S1–S7 and Tables S1–S12.
