# Supplementary material for: Integrated In Silico Prioritization of Antidiabetic Phytochemicals from Uvaria chamae P. Beauv. Based on Docking, Induced-Fit Docking, QSAR, and ADMET Analyses
Source: Molecules. 2026 May 29;31(11):1879. doi: 10.3390/molecules31111879 (PMC13257571; doi:10.3390/molecules31111879)
Supplement: Supplementary file 1 [file molecules-31-01879-s001.zip › molecules-4284169-supplementary.pdf]

Supplementary Information

Integrated In Silico Prioritization of Antidiabetic Phytochemicals from *Uvaria chamae* P. Beauv. Based on Docking, Induced-Fit Docking, QSAR, and ADMET Analyses

Toussaint Sovegnon [1,2,3], Sèdami Medegan Fagla [4], Brice Boris Legba [1], Joseph Lorent [2], Joelle Quetin-Leclercq [5], Habib Ganfon [3], Jean-Robert Klotoe [1], Fernand Gbaguidi [6] and Victorien Dougnon [1,*]

[1] Research Unit in Applied Microbiology and Pharmacology of Natural Substances, Research Laboratory in Applied Biology, Polytechnic School of Abomey-Calavi, University of Abomey-Calavi, Abomey Calavi P.O. Box 526, Benin; semevo.sovegnon@uclouvain.be (T.S.); legba.boris5@gmail.com (B.B.L.); jrklotoe@yahoo.fr (J.-R.K.)

[2] Department of Cellular and Molecular Pharmacology, Louvain Drug Research Institute, UCLouvain, Avenue E. Mounier, 73, B1.73.05, 1200 Brussels, Belgium; joseph.lorent@uclouvain.be

[3] Laboratory of Pharmacognosy, Faculty of Health Sciences, University of Abomey-Calavi, 01, Cotonou P.O. Box 188, Benin; habib.ganfon@uac.bj

[4] Medicinal and Organic Chemistry Laboratory, Faculty of Health Sciences, University of Abomey-Calavi, 01, Cotonou P.O. Box 188, Benin; smedeganfagla@presidence.bj

[5] Pharmacognosy Research Group, Louvain Drug Research Institute, UCLouvain, Avenue E. Mounier, 72, B1.72.03, 1200 Brussels, Belgium; joelle.leclercq@uclouvain.be

[6] National Laboratory of Pharmacognosy, Beninese Center for Scientific and Technical Research, 01, Oganla, Porto-Novo P.O. Box 06, Benin; ahokannou@yahoo.fr

* Correspondence: victorien.dougnon@gmail.com

Methods :

Table S1: Docking grid parameters used for molecular simulations

ID PDB	*co-crystallized ligand*	*Center of the grid (X)*	*Center of the grid (Y)*	*Center of the grid (Z)*
2Q5S	5-chloro-1-(4-chlorobenzyl)-3-(phenylthio)-1h-indole-2-carboxylic acid	17.53	19.58	9.12
2PRG	Rosiglitazone	49.72	-36.98	19.29
1B2Y	Alpha ascarbose	18.91	5.79	47.01
3L2M	α-cyclodextrin	46.99	38.04	71.79
2QBQ	4-Bromo-3-(Carboxymethoxy)-5-{3-[(3,3,5,5-Tetramethylcyclohexyl)amino]phenyl}thiophene-2-Carboxylic Acid	47.36	11.51	2.11
3C45	(2S,3S)-3-{3-[2-chloro-4-(methylsulfonyl)phenyl]-1,2,4-oxadiazol-5-yl}-1-cyclopentylidene-4-cyclopropyl-1-fluorobutan-2-amine	39.67	49.26	38.34
2QMJ	Acarbose	-20.83	-6.69	-5.16
3K35	Adp-ribose	22.82	3.82	6.2

Result :

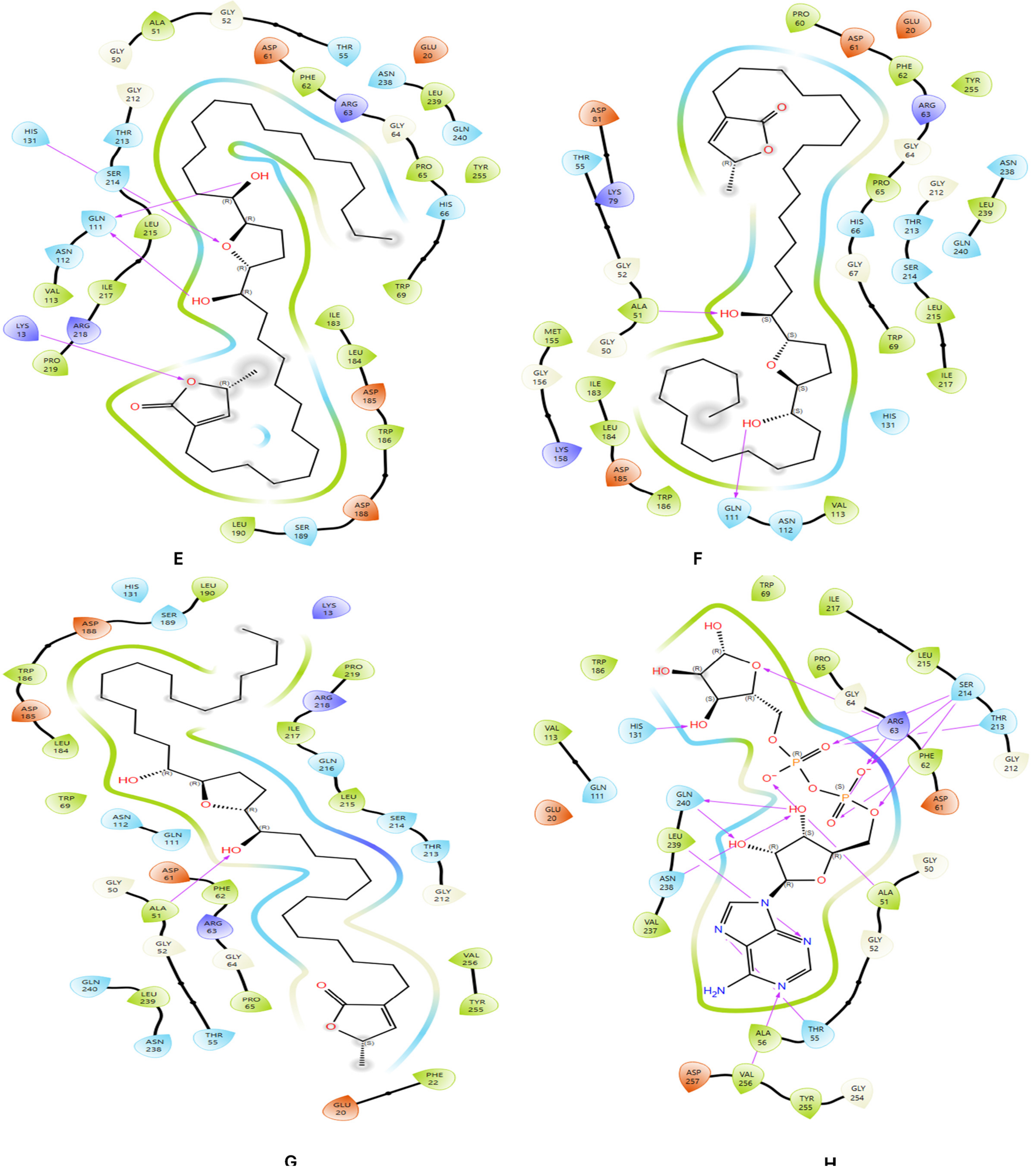

Figure S1. Two-dimensional ligand–protein interaction maps of selected compounds with human SIRT6 (PDB ID: 3K35). E.) Cis-uvariamicin-I interaction; F.) Uvarimicin-II interaction; G.) Uvariamicin-I interaction; H.) Adp-ribose (standard) interaction

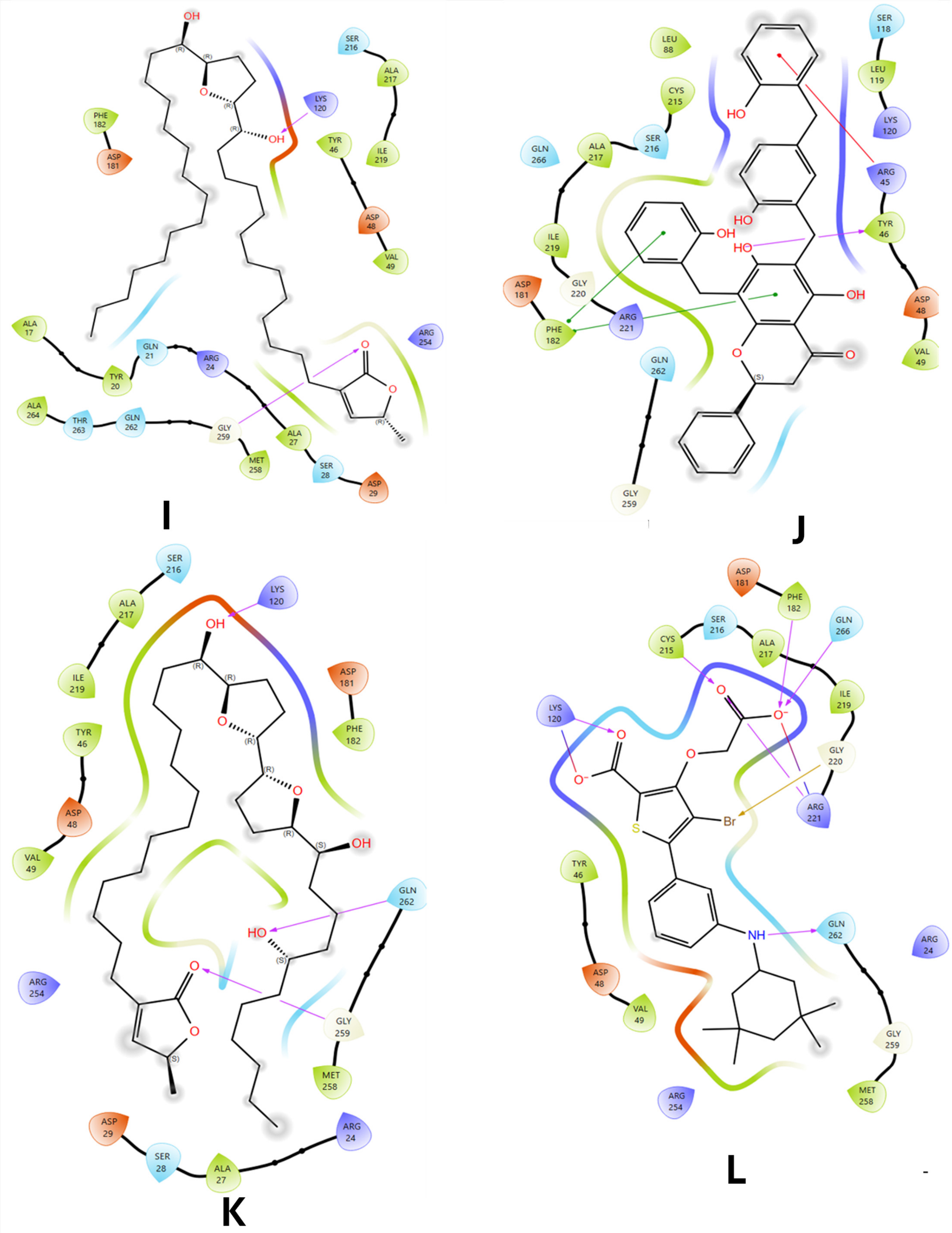

Figure S2. Two-dimensional ligand–protein interaction maps of selected compounds with protein tyrosine phosphatase 1B (PDB ID: 2QBQ). I.) Cis-Uvariamicin interaction; J.) Uvarinol interaction; K.) Squamocin interaction; L.) 4-Bromo-3-(Carboxymethoxy)-5-{3-[(3,3,5,5-Tetramethylcyclohexyl)amino]phenyl}thiophene-2-Carboxylic Acid (standard) interaction

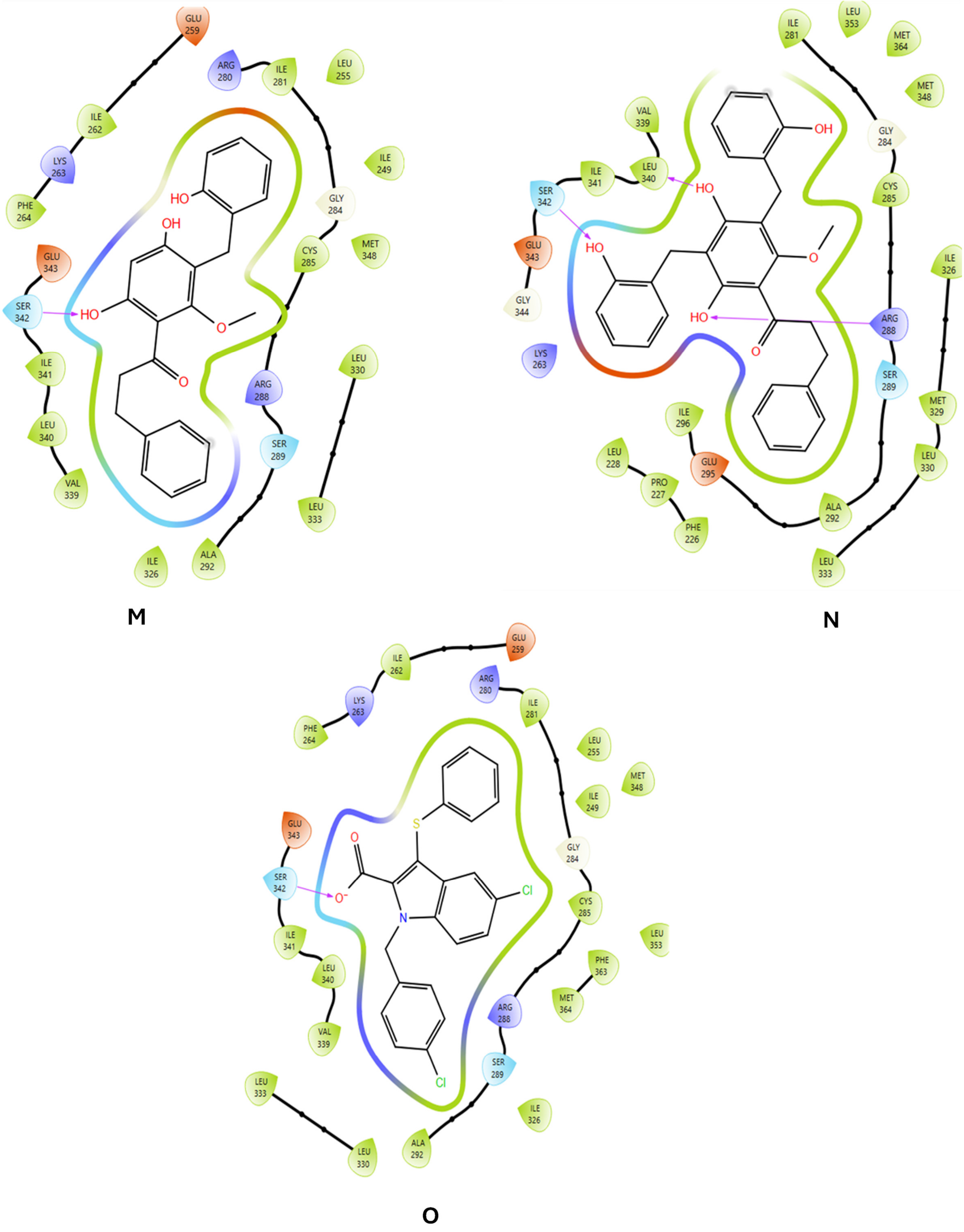

Figure S3. Two-dimensional ligand–protein interaction maps of selected compounds with PPARγ (PDB ID: 2Q5S). M.) Isouvaretin; N.) Diuvaretin interaction; O.) 5-chloro-1-(4-chlorobenzyl)-3-(phenylthio)-1h-indole-2-carboxylic acid (standard) interaction

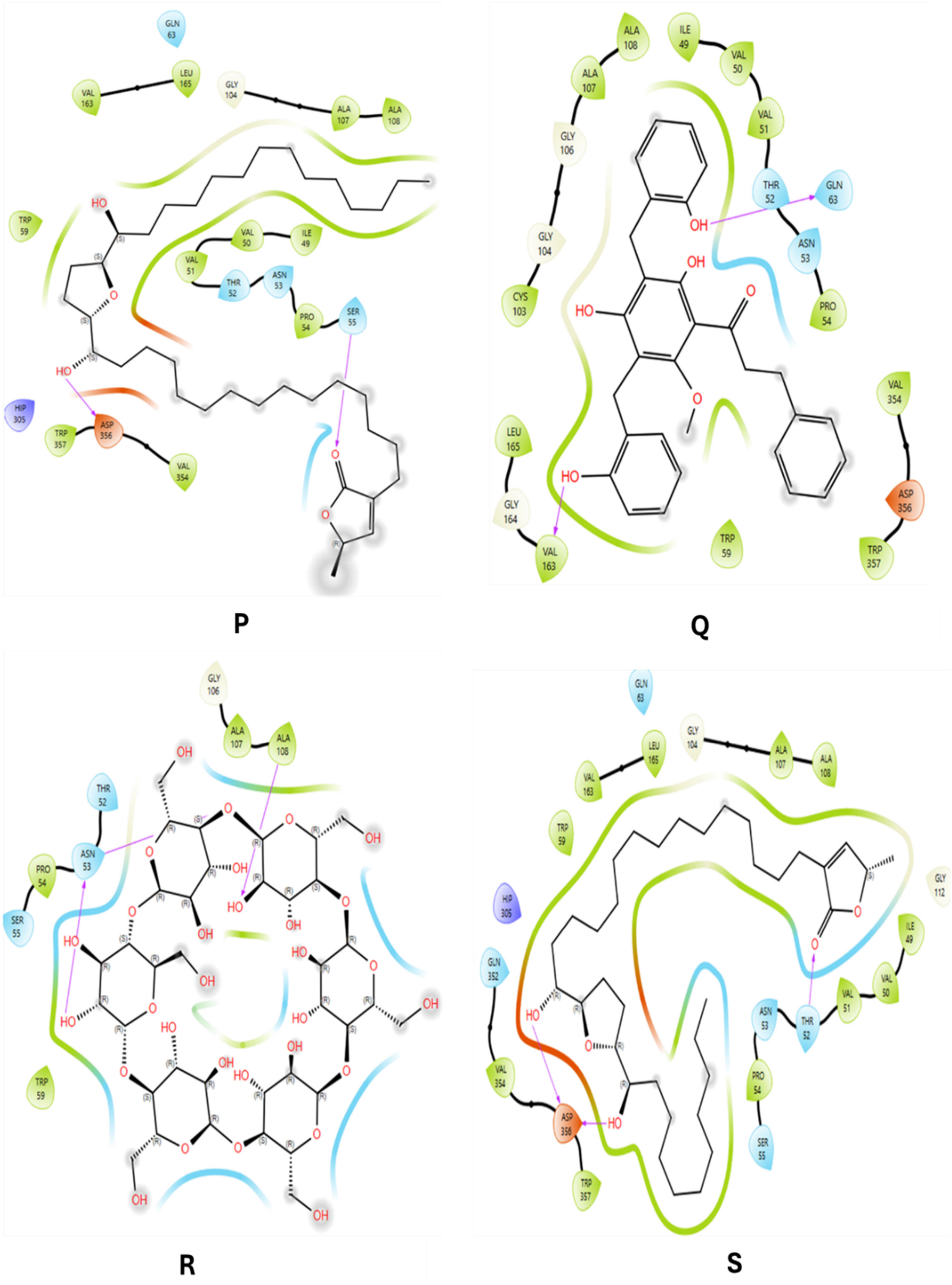

Figure S4. Two-dimensional ligand–protein interaction maps of selected compounds with pig pancreatic α-amylase (PDB ID: 3L2M). P.) Uvariamicin II interaction; Q.) Diuvaretin interaction; R.) Alpha cyclodexrin (standard insteraction); S.) Annotemoyin-1 interaction

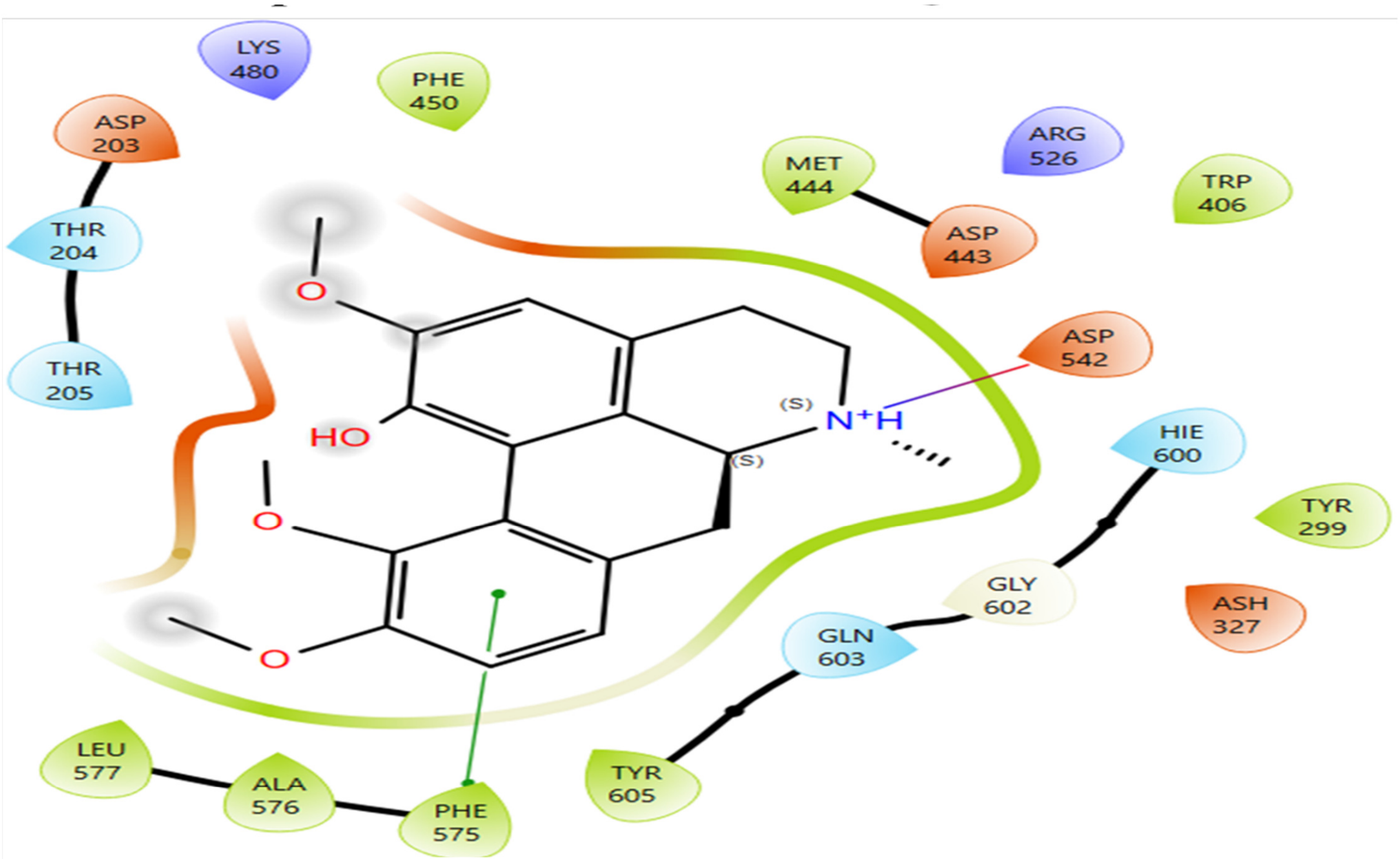

T

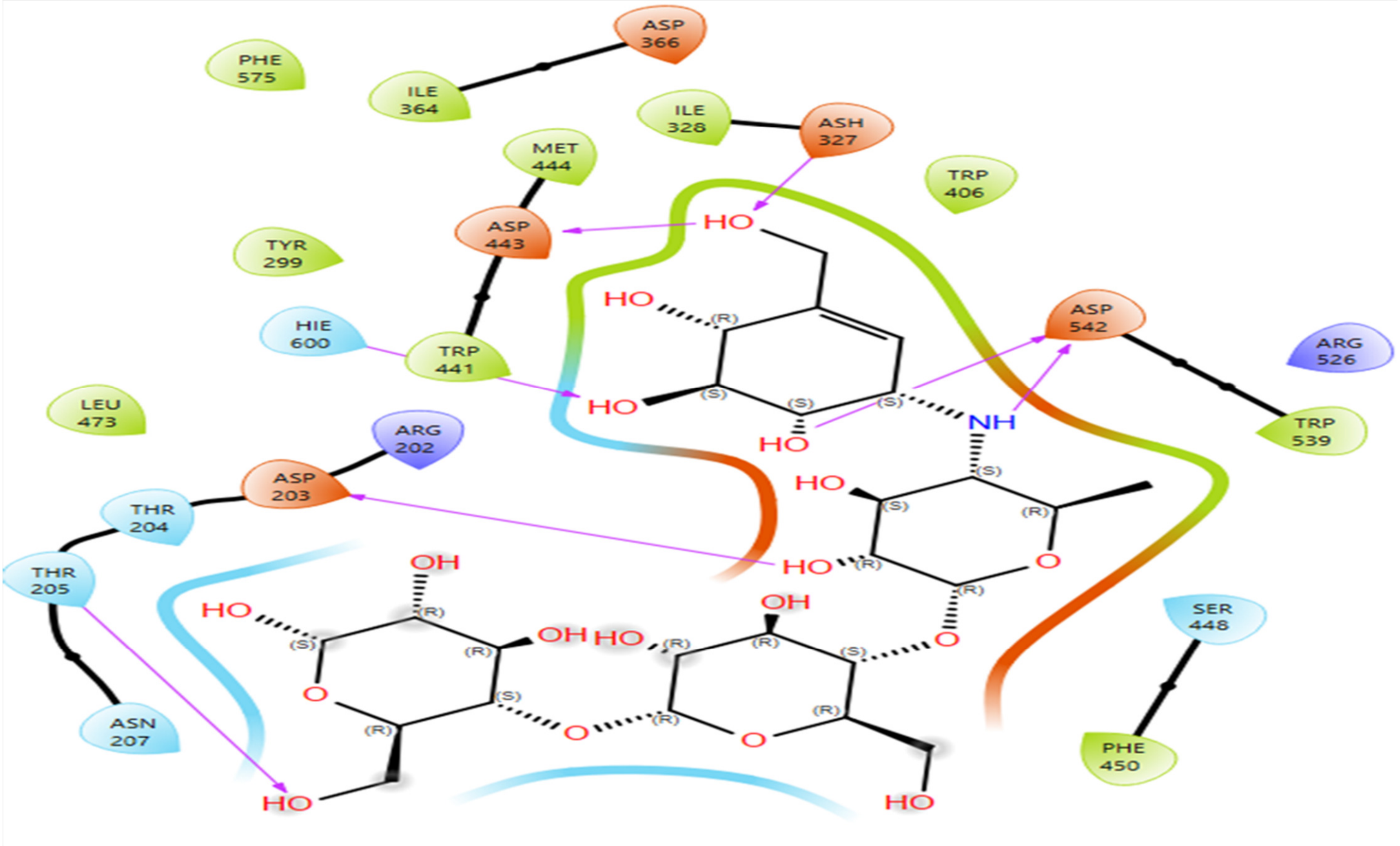

U

Figure S5. Two-dimensional ligand–protein interaction maps of selected compounds with N-terminal human maltase-glucoamylase (PDB ID: 3L4Z) T.) Corydine interaction; U.) Alpha acarbose (standard) interaction

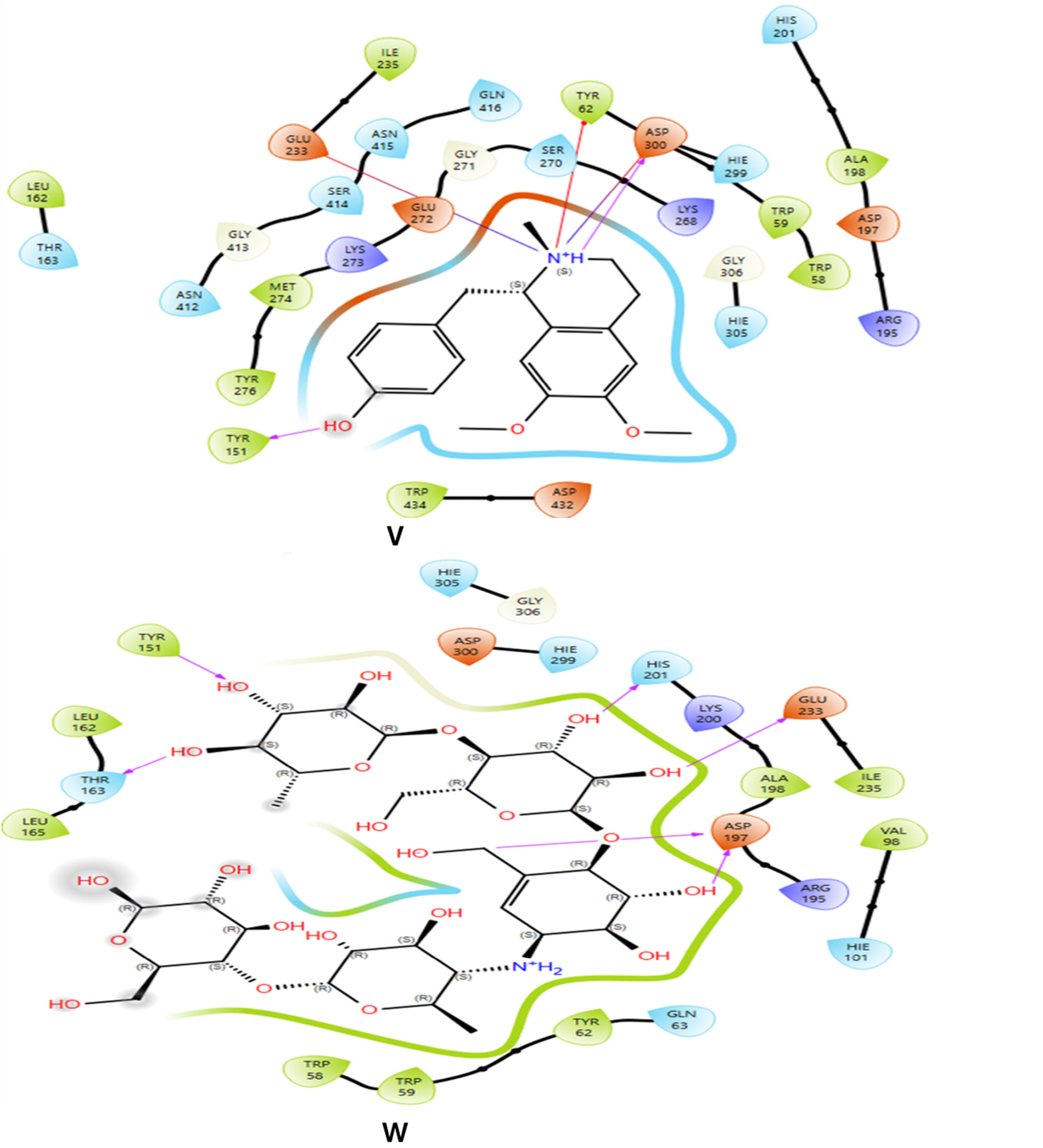

Figure S6. Two-dimensional ligand–protein interaction maps of selected compounds with human pancreatic α-amylase (PDB ID: 1B2Y). V.) (+)-Armepavine interaction; W.) Alpha acarbose (standard) interaction

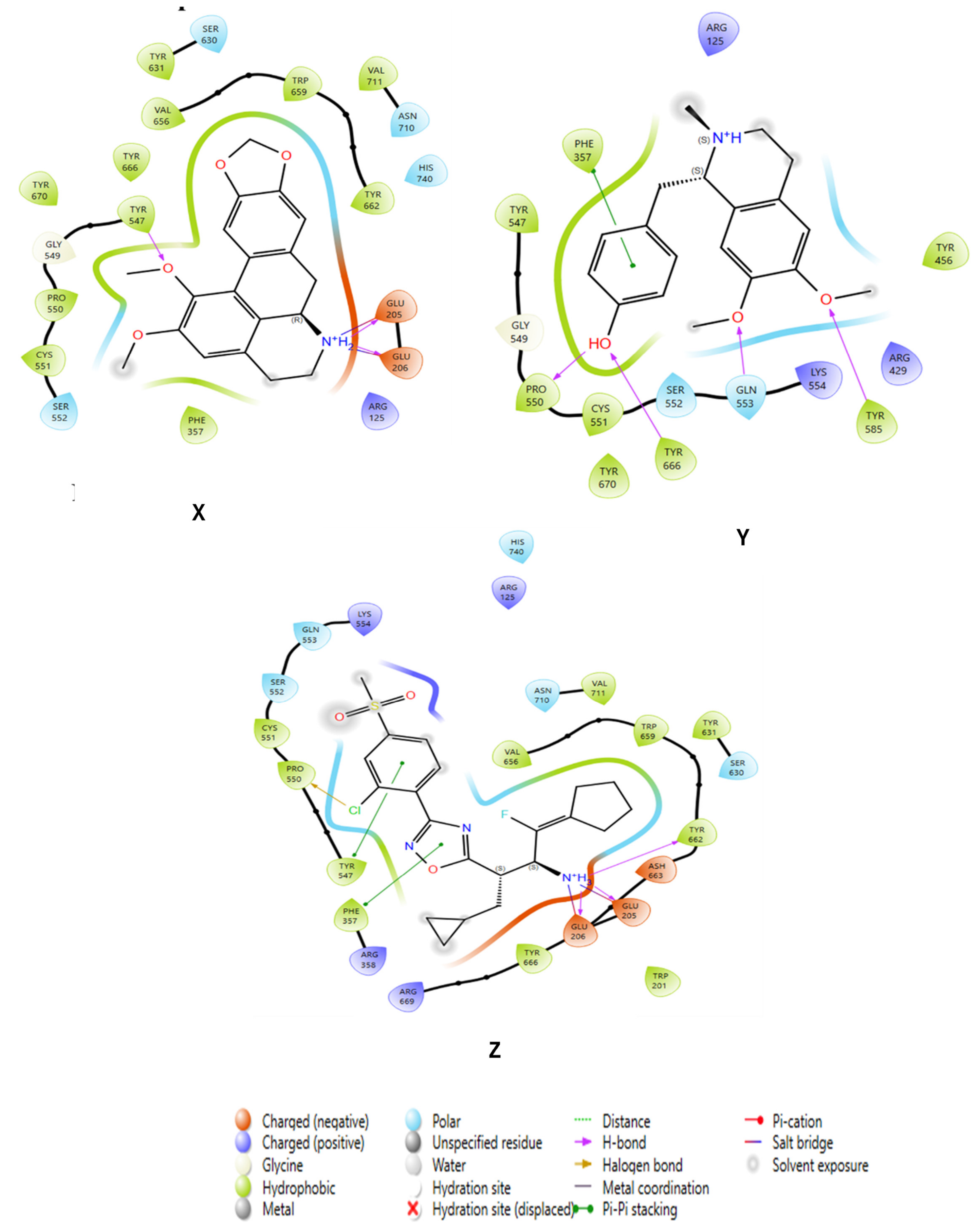

Figure S7. Two-dimensional ligand–protein interaction maps of selected compounds with dipeptidyl peptidase-4 (DPP-4/CD26) (PDB ID: 3C45). X.) Nornanterin interaction; Y.) (+)- Armepavin Interaction; Z.) (2S,3S)-3-{3-[2-chloro-4-(methylsulfonyl)phenyl]-1,2,4-oxadiazol-5-yl}-1-cyclopentylidene-4-cyclopropyl-1-fluorobutan-2-amine (Standard) interaction

Table S2: ADMET Properties

Class	Properties	Isouvaretin	Diuvaretin	Solamin	O,O-Diméthylcoclaurine	cis-UvariamicinI	Uvarinol	Squamocin	Nornanternine	(+)-Armepavine	Uvariamicin II	Uvariamicin-I	Annotemoyin-1	Corydine	4-Bromo-3-(Carboxymethoxy)-5-{3-[(3,3,5,5-Tetramethylcyclohexyl)amino]phenyl}thiophene-2-Carboxylic Acid	Rosiglitazone	5-chloro-1-(4-chlorobenzyl)-3-(phenylthio)-1h-indole-2-carboxylic acid	Alpha acarbose	(2S,3S)-3-{3-[2-chloro-4-(methylsulfonyl)phenyl]-1,2,4-oxadiazol-5-yl}-1-cyclopentylidene-4-cyclopropyl-1-fluorobutan-2-amine	Adp-ribose	Alpha cyclodexrine
**Absorption**	Caco-2 Permeability	-4,956	-5.045	-5.103	-5.03	-5.138	-5,126	-5.184	-4.991	-4,494	-5.07	-5.067	-5.064	-5,012	-5.495	-4,986	-4,816	-7.048	-5.3	-6.023	-8.28
	MDCK Permeability	-4.763	-4.743	-4.849	-4.764	-4.836	-4.8	-4.766	-4.789	-4.621	-4.893	-4.878	-4.861	-4.77	-5.014	-4.506	-4.812	-4.96	-4.933	-5.109	-4.654
	Pgp-inhibitor	+++	+++	---	+++	---	--	---	---	++	---	---	---	---	---	++	+++	---	---	---	---
	Pgp-substrate	---	---	+	+	---	---	+++	-	++	---	--	--	-	---	---	---	+++	---	---	+++
	HIA	---	---	+++	--	+++	+	---	---	---	+++	+++	++	--	---	---	---	+++	---	++	++
	F20%	--	-	+++	--	+++	+	+++	---	+++	+++	+++	+++	++	---	---	---	+++	---	---	---
	F30%	+++	+++	+++	---	+++	+++	+++	---	+	+++	+++	+++	++	---	---	-	+++	---	+++	++
	F50%	+++	+++	+++	+	+++	+++	+++	+	+++	+++	+++	+++	+++	--	---	-	+++	---	---	+++
**Distribution**	PPB (%)	98,20%	98.1%	100.2%	64.1%	99.6%	98,30%	95.8%	66.2%	49,30%	100.7%	100.7%	100.0%	78,90%	98.7%	99,50%	99,30%	15.9%	88.0%	27.2%	-8.2%
	VD	2,387	0.384	29.422	0.103	1.399	2,168	0.372	0.476	1,532	1.641	1.662	1.481	1.18	0.646	0,235	0,617	-0.527	0.031	-0.479	-0.451
	BBB Penetration	---	---	---	---	---	---	---	-	-	---	---	---	--	---	---	+++	---	--	---	---
	Was (%)	1.20%	1.6%	0.7%	27.5%	1.0%	1%	3.4%	35.7%	45,30%	0.6%	0.6%	0.8%	25,30%	1.1%	0,30%	0.2%	94.3%	9.2%	59.3%	119.3%
**Metabolism**	CYP1A2 inhibitor	+++	---	--	---	---	---	---	+	---	---	-	+++	+	---	++	---	---	---	---	---
	CYP1A2 substrate	+++	+++	---	+++	---	---	---	+++	+++	---	---	---	+++	---	---	---	---	---	---	---
	CYP2C19 inhibitor	--	+++	+++	---	+	+++	---	---	---	---	++	+++	---	---	---	---	---	--	---	---
	CYP2C19 substrate	--	---	---	+++	+	---	---	+++	+++	--	---	---	+++	---	--	---	---	---	---	---
	CYP2C9 inhibitor	+++	+++	-	---	---	+++	---	---	---	---	---	---	---	---	--	+++	---	++	---	---
	CYP2C9 substrate	+++	+++	+	+++	+++	+++	--	+++	++	+++	+++	+++	++	---	+++	+++	---	-	---	---
	CYP2D6 inhibitor	+	---	---	+++	---	---	---	+++	---	---	--	--	---	---	---	---	---	--	---	---
	CYP2D6 substrate	+++	++	---	+++	-	+++	---	+++	+++	+	---	+	+++	---	-	---	---	---	---	---
	CYP3A4 inhibitor	+++	-	--	--	++	-	---	++	-	++	-	++	---	---	-	---	---	---	---	---

	CYP3A4 substrate	+	+++	---	+++	--	+++	---	+	++	---	---	---	+++	--	+++	--	---	--	---	---
**Excretion**	CL	4,851	7.768	4.618	10.33	4,984	4,764	5.254	5,574	12,003	4,585	4,711	4,905	5,338	2,564	6,515	0.7	0.005	3,397	1,389	-2.143
	T1/2	1.101	0.912	2.118	1,835	2.475	1,232	1.317	1,767	2,337	2.637	2.299	1.773	2,918	1.258	0.928	1.455	3.813	0.688	2.371	6.777
**Toxicity**	hERG Blockers	0,126	0.103	0.676	0.504	0.866	0,115	0.728	0.605	0,537	0.734	0.694	0.627	0,406	0.033	0.164	0,707	0.001	0.508	0.012	0.0
	DILI	0,028	0.019	0.311	0.588	0.14	0,053	0.1	0.431	0,017	0.119	0.295	0.309	0,053	0.997	0.959	1.0	0.756	0.998	0.998	1.0
	AMES Toxicity	0,3	0.24	0.301	0.693	0.049	0,494	0.369	0.835	0,343	0.033	0.326	0.395	0,656	0.135	0.291	0,213	0.906	0.129	0.327	1.0
	Rat Oral Acute Toxicity	0,192	0.157	0.256	0.376	0.204	0,253	0.228	0.784	0.837	0.067	0.276	0.293	0,727	0.365	0.258	0.15	0.001	0.254	0.028	0.0
	FDAMDD	0.39	0.465	0.861	0.775	0.594	0.55	0.934	0.895	0.974	0.508	0.841	0.831	0,899	0.181	0.597	0,509	0.001	0.789	0.979	0.0
	Skin Sensitization	0,846	0.906	1.0	0.486	1.0	0,964	1.0	0.905	0,468	1.0	1.0	1.0	0,53	0.324	0.878	0,999	0.999	0.118	0.999	1.0
	Carcinogenicity	0,216	0.15	0.522	0.343	0.096	0,111	0.402	0.57	0,198	0.185	0.52	0.548	0,755	0.167	0.266	0,195	0.004	0.281	0.411	0.0
	Eye Corrosion	0,134	0.028	0.339	0.011	0.001	0	0.076	0.002	0,009	0.434	0.227	0.231	0,001	0.0	0.0	0	0.0	0.0	0.0	0.0
	Eye Irritation	0,977	0.959	0.906	0.843	0.269	0,961	0.571	0.11	0,725	0.963	0.9	0.898	0,172	0.445	0.058	0,218	0.007	0.009	0.29	0.0
	Respiratory Toxicity	0,944	0.978	0.974	0.683	0.797	0,962	0.975	0.984	0,934	0.948	0.97	0.957	0,955	0.156	0.864	0.623	0.001	0.493	0.923	0.0

Table S3: Physicochemical properties and medicinal chemistry parameters of the compounds

Parameter	Isouvaretin	Diuvaretin	Solamin	O,O-Diméthylcoclaurine	cis-Uvariamicin I	Uvarinol	Squamocin	Annotemoyin-1	Nornanternine	(+)-Armépavine	Uvariamicin II	Uvariamicin-I	Corydine	Rosiglitazone	4-Bromo-3-(Carboxymethoxy)-5-{3-[(3,3,5,5-Tetramethylcyclohexyl)amino]phenyl}thiophene-2-Carboxylic acid	5-chloro-1-(4-chlorobenzyl)-3-(phenylthio)-1h-indole-2-carboxylic acid	Alpha acarbose	(2S,3S)-3-{3-[2-chloro-4-(methylsulfonyl)phenyl]-1,2,4-oxadiazol-5-yl}-1-cyclopentylidene-4-cyclopropyl-1-fluorobutan-2-amine	Adp-ribose	Alpha cyclodextrine
**PHYSIOCHEMCAL PROPERTIES**																				
**Molecular Weight**	378,15	484.19	578,49	313.17	606,52	574.2	622.48	564.48	325.13	313,17	606.52	592.51	341,16	357.11	509.09	427,02	645.25	453.13	559.07	972.32
**Van der Waals volume**	398,281	511,678	652 777	333,059	687,369	599,221	679,097	635,481	324,737	333,059	687,369	670,073	350 589	350,993	454.98	403.35	573,315	420,876	440,926	835,024
**nHA**	5.0	6.0	5.0	4.0	5.0	7.0	7.0	5.0	5.0	4.0	5.0	5.0	5.0	6.0	6.0	3.0	19.0	6.0	19.0	30.0
**nHD**	3.0	4.0	2.0	1.0	2.0	5.0	3.0	2.0	1.0	1.0	2.0	2.0	1.0	1.0	3.0	1.0	14.0	2.0	9.0	18.0
**nRot**	7.0	9.0	27.0	5.0	29.0	7.0	25.0	26.0	2.0	4.0	29.0	28.0	3.0	7.0	7.0	5.0	9.0	7.0	9.0	6.0
**nRing**	3.0	4.0	2.0	3.0	2.0	6.0	3.0	2.0	5.0	3.0	2.0	2.0	4.0	3.0	3.0	4.0	4.0	4.0	4.0	22.0
**MaxRing**	6.0	6.0	5.0	10.0	5.0	10.0	5.0	5.0	19.0	10.0	5.0	5.0	16.0	6.0	6.0	9.0	6.0	6.0	9.0	0.0
**nHot**	5.0	6.0	5.0	4.0	5.0	7.0	7.0	5.0	5.0	4.0	5.0	5.0	5.0	7.0	8.0	6.0	19.0	9.0	21.0	30.0
**fChar**	0	0.0	0	0.0	0	0	0.0	0.0	0.0	0	0.0	0.0	0	0	0.0	0	0.0	0.0	0.0	0.0
**nRig**	19.0	25.0	11.0	17.0	11.0	36.0	16.0	11.0	24.0	17.0	11.0	11.0	20.0	19.0	19.0	23.0	24.0	22.0	22.0	48.0
**Flexibility = n Rot /nRig**	0,368	0.36	2,455	0.294	2,636	0,194	1.562	2.364	0.083	0,235	2.636	2.545	0,15	0,368	0.368	0,217	0.375	0.318	0.409	0.125
**Stereo Centers**	0	0.0	5.0	1.0	5.0	1.0	8.0	5.0	1.0	1.0	5.0	5.0	1.0	1.0	0.0	0	19.0	2.0	10.0	30.0
**TPSA**	86,99	107.22	75,99	39.72	75,99	127,45	105.45	75.99	48.95	41,93	75.99	75.99	51.16	71,53	95.86	42,23	321.17	99.08	291.52	474.9
**logS**	-3,31	-3.059	-6,063	-2.941	-5,902	-3,491	-4.982	-5.631	-3.097	-2,714	-6.71	-6.248	-2,677	-4,112	-4.285	-4,533	0.364	-4.136	-0.787	0.948
**logP**	3,732	4.629	8.46	2.268	9.026	5,772	5.254	7.862	2.162	2.113	8.948	8.401	2.017	2,667	4.93	5,638	-4.506	2.818	-3.161	-5.335
**logD**	3,169	3.314	4.74	2.582	4.903	3,802	3.734	4.506	2.407	2.026	4.832	4.664	2.209	2,806	3.004	3,756	-3.311	2.784	-1.302	-4.288
**MEDICINAL CHEMISTRY**																				

**QED**	0.536	0.233	0,075	0.92	0,065	0,146	0.07	0.081	0.92	0,941	0.065	0.07	0,929	0,821	0.402	0,378	0.103	0.646	0.14	0.117
**SAscore**	Easy	Easy	Facile	Easy	Facile	Facile	Easy	Easy	Easy	Facile	Easy	Easy	Facile	Facile	Easy	Facile	Easy	Easy	Easy	Hard
**Fsp3**	0.174	0.167	0,917	0.368	0,921	0,139	0.919	0.914	0.368	0,368	0.921	0.919	0,4	0,278	0.478	0,045	0.92	0.524	0.667	1.0
**MCE-18**	18.0	24.0	27.884	51.692	27.781	105.317	42.817	27.94	84.615	54,923	27.781	27.831	75.571	49.391	56.471	22.0	89.333	85.938	109.2	144.5
**NPscore**	0.882	0.821	1,606	0.696	1,532	1,043	1.818	1.647	1.613	0,89	1.532	1.568	1.501	-0,929	-0.519	-1,08	1.829	-1.135	1.266	0.522
**Lipinski Rule**	Accepted	Accepted	Rejected	Accepted	Rejeté	Rejeté	Rejected	Rejected	Accepted	Accepted	Rejected	Rejected	Accepted	Accepted	Accepted	Accepted	Rejected	Accepted	Rejected	Rejected
**Pfizer Rule**	Accepted	Accepted	Accepted	Accepted	Accepted	Accepted	Accepted	Accepted	Accepted	Accepted	Accepted	Accepted	Accepted	Accepted	Accepted	Rejected	Accepted	Accepted	Accepted	Accepted
**GSK Rule**	Accepted	Rejected	Rejected	Accepted	Rejeté	Rejeté	Rejected	Rejected	Accepted	Accepted	Rejected	Rejected	Accepted	Accepted	Rejected	Rejected	Rejected	Rejected	Rejected	Rejected
**Golden Triangle**	Accepted	Accepted	Rejected	Accepted	Rejeté	Rejeté	Rejected	Rejected	Accepted	Accepted	Rejected	Rejected	Accepted	Accepted	Rejected	Accepted	Rejected	Accepted	Rejected	Rejected
**PAINS**	0	0	0	0	0	0	0	0	0	0	0	0	0	0	0	0	0	0	0	0
**ALARM NMR**	2	2	2	1	2	2	2	2	0	1	2	2	1	5	3	2	0	1	0	0
**BMS**	0	0	1	0	1	2	1	1	0	0	1	1	0	0	0	0	1	0	1	1
**Chelator Rule**	0	0	0	0	0	0	0	0	0	0	0	0	1	0	0	1	0	0	1	0

Table S5. Ranking of compounds targeting protein 3L2M based on Binding energy (Kcal/mol)s and MM/GBSA binding energies

Ranking	Pubchem/Coconut ID	Binding energy (Kcal/mol)	MMGBSA dG Bind (Kcal/mol)
**1.**	101392149	-4,681359988	-62,08533239
**2.**	3085222	-6,090449662	-57,5276637
**3.**	CNP0240351,2	-4,020467024	-56,44155779
**4.**	441612	-5,352757248	-55,42548827
**5.**	CNP0165224,1	-3,504998311	-51,28292632
**6.**	100418	-5,375246267	-49,86037861
**7.**	CNP0150614,1	-3,938728528	-48,86948084
**8.**	65981	-4,290722274	-47,51073032
**9.**	108065	-6,421935106	-47,17476282
**10.**	131753021	-1,547159168	-47,15838382
**11.**	65981	-3,140535713	-46,95465925
**12.**	11376469	-3,90233159	-46,69128114
**13.**	CNP0239276,2	-3,029000488	-46,12270575
**14.**	21721823	-4,536195313	-45,90256775
**15.**	3084228	-4,036720684	-44,22299894
**16.**	680292	-3,601557583	-43,98676146
**17.**	638072	-3,126347761	-43,37280247
**18.**	4369359	-4,489102464	-43,36946015
**19.**	10829011	-3,213853369	-42,73447782
**20.**	4369359	-5,199512039	-42,56723499
**21.**	445421	-8,620515621	-42,35944337
**22.**	76315048	-3,435704641	-41,39323876
**23.**	108065	-3,821633176	-40,73004417
**24.**	100418	-2,325742188	-40,31257698
**25.**	21721821	-3,87420515	-39,99920419
**26.**	108065	-3,867965267	-39,84312527
**27.**	3085222	-3,496013234	-38,89800725
**28.**	21721823	-4,407715868	-38,52878328
**29.**	21721823	-4,406594605	-38,51349443
**30.**	73447	-5,045203926	-37,95453014
**31.**	68071	-3,821892406	-37,94916039
**32.**	100418	-4,175614858	-37,85026824
**33.**	3084228	-3,320229668	-37,24267933
**34.**	74778	-0,922908364	-36,50902891
**35.**	10829011	-3,019809027	-36,42827953
**36.**	181193	-5,459282293	-36,06815597
**37.**	73201	-5,330017356	-35,8369416
**38.**	127149	-2,040792854	-35,6054536

**39.**	44577080	-2,38774887	-34,72617572
**40.**	444913	-7.87	-34,61
**41.**	151670	-2,366537207	-33,31458244
**42.**	222284	-2,907896278	-32,79355246
**43.**	3085222	-4,172750016	-32,71329693
**44.**	108065	-4,766872737	-32,44244846
**45.**	5352860	-2,25360959	-31,67941906
**46.**	44577080	-2,660169385	-31,13491541
**47.**	5318528	-3,716834414	-30,17463257
**48.**	5281855	-3,126859656	-29,94593431
**49.**	680292	-2,740647536	-29,28779667
**50.**	CNP0165224,1	-3,290936672	-29,20185081
**51.**	100418	-3,352903899	-29,09339365
**52.**	5280435	-1,828502061	-29,07984747
**53.**	101687121	-2,600316652	-28,68074071
**54.**	3085222	-4,189946388	-28,49106652
**55.**	73447	-2,481976575	-28,46551528
**56.**	151670	-4,435202415	-28,30764257
**57.**	21721821	-2,504631322	-28,26177468
**58.**	6483649	-4,516872115	-28,25407378
**59.**	68071	-1,378882481	-28,25183238
**60.**	3085222	-3,089259045	-28,12569412
**61.**	151670	-2,306806827	-28,04510302
**62.**	68071	-2,010752324	-27,67097071
**63.**	17697	-0,634353613	-27,35914436
**64.**	CNP0157012,2	-4,334409772	-27,32804672
**65.**	5318528	-1,676535581	-27,30572817
**66.**	CNP0157012,2	-3,535000535	-26,90675085
**67.**	6918391	-0,979220176	-26,81710823
**68.**	11230	-3,391562401	-26,70648828
**69.**	5281516	-0,306856333	-26,69524448
**70.**	441005	-2,126289982	-26,45485071
**71.**	5284507	-1,316183449	-26,41651931
**72.**	73447	-3,924097774	-26,40009172
**73.**	44577080	-0,955887688	-26,11535697
**74.**	6429080	-3,216238376	-25,06736474
**75.**	17100	-3,257001156	-24,88285353
**76.**	445421	-8,117778351	-24,86512074
**77.**	31253	-1,534052326	-24,85198659
**78.**	159846	-3,75899367	-24,76077774
**79.**	21721821	-4,148013951	-24,63767721
**80.**	18756	-1,783247736	-24,63062242
**81.**	5318528	-3,506608495	-24,48839753
**82.**	94164	-2,107571704	-24,36406713
**83.**	6549	-2,625107305	-24,33364034

**84.**	2345	-3,094248034	-24,29674335
**85.**	21721821	-2,859374742	-24,08029507
**86.**	985	1,595708171	-23,96654924
**87.**	9548705	-2,099027529	-23,46288502
**88.**	8794	-3,451144237	-23,08359371
**89.**	162952798	-2,991721903	-22,44497123
**90.**	7463	-2,911966029	-22,19618979
**91.**	12313020	-1,981254492	-21,95584765
**92.**	7461	-2,752219668	-21,90592557
**93.**	6430869	-2,660152744	-21,8755139
**94.**	5280794	-3,27206468	-21,84242098
**95.**	5281520	-2,296283037	-21,48451172
**96.**	68071	-2,945856004	-21,30431909
**97.**	442359	-2,157514803	-21,19688813
**98.**	5054	-3,710928653	-21,13699797
**99.**	22311	-2,219122464	-20,91125449
**100**	12303902	-2,937346263	-20,73397485
**101**	5281519	-2,086411084	-20,49046275
**102**	5318528	-1,872179059	-20,48980601
**103**	6483649	-2,791514762	-20,46582447
**104**	92138	-2,785088409	-20,27066928
**105**	18756	-1,559417177	-20,16381111
**106**	80208	-1,822761676	-20,00708779
**107**	7460	-2,976013714	-19,92819696
**108**	7462	-2,531487515	-19,13199152
**109**	73447	-3,792621825	-19,10637976
**110**	689043	-3,058860633	-18,6207008
**111**	21721821	-3,171906623	-18,19708094
**112**	6483649	-2,767534309	-18,13645275
**113**	13894537	-1,918925511	-17,87018905
**114**	5318528	-3,451569795	-17,76393589
**115**	91747125	-2,153037228	-17,54065439
**116**	151670	-5,435876137	-17,53611191
**117**	240	-2,700221433	-17,27534061
**118**	68071	-1,995955644	-17,26173777
**119**	4091	0,714652318	-17,08467306
**120**	181193	-2,881188415	-15,92597948
**121**	181193	-1,855360064	-15,85460781
**122**	73201	-0,004365385	-15,33130829
**123**	4091	-2,091425212	-14,58488031
**124**	21721823	-1,21769548	-14,57349958
**125**	10704181	-1,122648076	-12,3999972
**126**	4091	0,531839924	-11,01806789
**127**	5281855	-3,999681679	-9,662195221
**128**	100418	-0,791542193	-8,84468314

**129**	21721823	-4,160348951	-7,371382875
**130**	5281855	-2,121061713	-6,082100486
**131**	5281855	-3,120063955	-5,803810377
**132**	5054	-0,400693532	-4,852433297
**133**	689043	-0,637115493	0,740864018
**134**	689043	-1,412962182	3,730236786

Table S6. Ranking of compounds targeting protein 3C45 based on Binding energy (Kcal/mol)s and MM/GBSA binding energies

Ranking	Pubchem/Coconut ID	Binding energy (Kcal/mol)	MMGBSA dG Bind (Kcal/mol)
**1.**	445421	-11,43897705	-69,65035221
**2.**	3084228	-8,342118482	-62,06615333
**3.**	317	-9,177	-58,03
**4.**	680292	-6,035266355	-55,1793328
**5.**	4369359	-9,3467624	-53,72648787
**6.**	444913	-12,06671086	-52,56473974
**7.**	680292	-5,455529163	-50,62170395
**8.**	73447	-7,408692604	-43,06345408
**9.**	CNP0239276,2	-0,471880654	-42,92133677
**10.**	10829011	-4,898169444	-39,03411349
**11.**	11376469	-4,565403208	-37,44010124
**12.**	10829011	-3,727742964	-37,0327389
**13.**	65981	-4,263808954	-35,24294621
**14.**	5280435	-3,717956751	-34,99044378
**15.**	4369359	-5,932230502	-34,60007201
**16.**	3084228	-5,264433575	-34,18036503
**17.**	101392149	-4,350312987	-33,98594372
**18.**	108065	-8,115713483	-33,61353986
**19.**	689043	-5,471745022	-33,51136628
**20.**	4091	-2,250961114	-32,46356614
**21.**	CNP0150614,1	-3,076175634	-31,9311077
**22.**	44577080	-4,19363171	-31,8707591
**23.**	CNP0165224,1	-5,360444301	-31,0150226
**24.**	131753021	-5,949658148	-29,690679
**25.**	5318528	-4,04864639	-29,51386992
**26.**	3085222	-7,402705993	-28,90822049
**27.**	4091	-2,326454557	-27,66554797
**28.**	8794	-5,499482675	-26,86554256
**29.**	CNP0240351,2	-3,395009904	-26,40978507
**30.**	151670	-7,131406135	-26,05682818
**31.**	74778	-1,996719681	-25,69130106
**32.**	100418	-4,405062984	-24,78405092
**33.**	65981	-3,929353093	-24,58528845

**34.**	3085222	-7,991854773	-24,01061194
**35.**	21721823	-6,664922511	-23,51818268
**36.**	5281855	-2,649569387	-23,40380916
**37.**	2345	-4,955539922	-22,78605779
**38.**	5281516	-3,134392157	-21,9471913
**39.**	638072	-4,823505881	-21,54360278
**40.**	240	-4,583594531	-21,08599446
**41.**	127149	-5,47263302	-20,99875649
**42.**	6483649	-6,594087302	-20,85722802
**43.**	317	-6,586228394	-20,77460167
**44.**	44577080	-1,685771372	-20,62517533
**45.**	108065	-5,31176915	-20,24441185
**46.**	17697	-2,122343173	-19,46411865
**47.**	80208	-4,30188484	-19,27464654
**48.**	108065	-5,887034865	-19,26944636
**49.**	100418	-7,291503676	-19,16090216
**50.**	101687121	-4,503941599	-19,01502615
**51.**	100418	-5,702588665	-18,18922234
**52.**	44577080	-3,397522297	-18,16637882
**53.**	159846	-4,354857454	-17,70969353
**54.**	18756	-3,634993538	-17,64916818
**55.**	21721823	-6,048819985	-16,69905445
**56.**	21721823	-6,72725319	-15,46972289
**57.**	31253	-3,493206703	-15,18445225
**58.**	5281519	-1,723853357	-15,07017279
**59.**	5054	-4,468510377	-15,02914666
**60.**	73201	-5,131523932	-14,91166019
**61.**	445421	-7,694878528	-14,37085906
**62.**	181193	-5,868265271	-13,44438026
**63.**	10398656	-4,694353321	-13,35081129
**64.**	5284507	-3,11008126	-13,27658831
**65.**	73447	-5,194609567	-13,12790832
**66.**	3085222	-7,495946423	-13,01002927
**67.**	76315048	-4,501955729	-12,99557759
**68.**	441612	-0,572854011	-12,69288271
**69.**	CNP0157012,2	-2,800848254	-12,66878864
**70.**	259846	-3,117697174	-12,49967026
**71.**	4091	-4,738670342	-11,85304978
**72.**	5352860	-3,851049387	-11,70642698
**73.**	5318528	-2,23590846	-11,05085314
**74.**	21721821	-3,007167519	-11,00038873
**75.**	44577078	-4,96467104	-10,9684534
**76.**	68071	-4,548406959	-10,88628428
**77.**	527113	-4,483823278	-10,4271392
**78.**	10798255	-2,709555532	-10,18583216
**79.**	92138	-2,678474346	-9,672943854

**80.**	CNP0157012,2	-2,674491998	-9,119957964
**81.**	442393	-3,673341842	-8,354445815
**82.**	7460	-4,085139265	-7,983245033
**83.**	5318528	-4,385948396	-7,655515353
**84.**	3085222	-5,711098106	-7,641771796
**85.**	11276107	-4,286422577	-7,375293223
**86.**	7463	-4,828688671	-7,22351309
**87.**	18756	-3,033231185	-6,824379049
**88.**	17100	-4,527737187	-6,768291767
**89.**	6918391	-4,045745538	-6,686125918
**90.**	CNP0165224,1	-3,480598483	-6,660930881
**91.**	21721821	-6,859713357	-6,630831865
**92.**	21721821	-4,65937646	-6,542104951
**93.**	181193	-6,46832605	-6,534312465
**94.**	441005	-4,027263214	-6,484102073
**95.**	689043	-3,558194714	-6,439643667
**96.**	19725	-2,333001917	-5,865741861
**97.**	7461	-4,245783838	-5,794070285
**98.**	22311	-4,2427057	-5,373732568
**99.**	6430869	-3,759113639	-5,032137492
**100.**	5281855	-4,163383138	-4,486507393
**101.**	9548705	-2,522934368	-4,144668378
**102.**	26049	-3,172077775	-3,9364952
**103.**	5281520	-1,730926179	-3,711927474
**104.**	160799	-4,058841729	-3,62495469
**105.**	21721823	-2,517906276	-3,547946384
**106.**	6549	-3,648636727	-3,324704523
**107.**	10438442	-2,711607026	-2,567234164
**108.**	73201	-3,024497826	-2,557860342
**109.**	ANP	-8,170463931	-2,487927281
**110.**	62566	-3,959416996	-2,415838582
**111.**	7462	-4,0192295	-2,337340104
**112.**	11230	-4,886122732	-1,63898127
**113.**	12046149	-3,463579018	-1,100523236
**114.**	12313020	-3,154618657	-1,082967488
**115.**	68071	-4,107487994	-1,073022152
**116.**	92231	-2,792149717	-0,951893347
**117.**	5280794	-3,943487078	-0,570210604
**118.**	68071	-5,04192167	-0,543552287
**119.**	162952798	-4,557420061	-0,320940062
**120.**	73447	-6,633058886	-0,247881692
**121.**	91747125	-2,42480663	-0,130217018
**122.**	5318528	-3,192183349	0,117348421
**123.**	68071	-3,665064552	0,427924079
**124.**	13894537	-2,385109633	1,54298052
**125.**	689043	-1,471276917	1,664389005

**126.**	5317570	-3,945552024	1,752441301
**127.**	1742210	-1,692227287	2,121892396
**128.**	6432221	-4,122400671	2,530920972
**129.**	6483649	-4,014260583	2,536458501
**130.**	12306047	-2,579870879	2,711052365
**131.**	93081	-3,379209726	2,750497132
**132.**	10704181	-1,607318973	2,760486817
**133.**	91753433	-3,718696569	3,372960685
**134.**	73447	-6,200214755	3,641408411
**135.**	151670	-6,174826936	3,937119083
**136.**	442359	-3,417858801	4,052390734
**137.**	91753170	-3,508969744	5,062714321
**138.**	2758	-2,882990634	5,638543321
**139.**	6429080	-4,356628834	5,829201763
**140.**	57339298	-3,431479181	7,835848598
**141.**	6483649	-3,973316372	8,592848813
**142.**	6616	-2,729045079	8,660438088
**143.**	12303902	-3,216510506	8,726474268
**144.**	3085222	-5,245210922	9,050222127
**145.**	101596917	-1,625212614	9,279830168
**146.**	985	-2,891634511	10,07949547
**147.**	151670	-5,845494274	10,13058861
**148.**	10899740	-1,509802302	10,18021231
**149.**	94164	-3,714300062	10,99067839
**150.**	108065	-4,604667982	11,00456552
**151.**	100418	-6,437867335	11,22671125
**152.**	68071	-4,538176025	11,66745162
**153.**	5318528	-4,061335408	11,67116513
**154.**	5281855	-2,692892416	12,12108745
**155.**	5281515	-2,311081809	14,21678621
**156.**	181193	-4,751376419	15,39999743
**157.**	64685	-2,253391589	16,15659062
**158.**	222284	-4,391082713	17,09781322
**159.**	21721821	-5,059253883	19,07315139
**160.**	100418	-5,326033743	19,56510779
**161.**	21721823	-5,378056108	23,04807445
**162.**	5281855	-2,798722515	30,62809948
**163.**	21721821	-4,10479197	36,3713349
**164.**	151670	-5,561529684	37,3080467
**165.**	5054	-1,500928403	47,28947915

Table S7. Ranking of compounds targeting protein 2QMJ based on Binding energy (Kcal/mol)s and MM/GBSA binding energies

Ranking	Pubchem/Coconut ID	Binding energy (Kcal/mol)	MMGBSA dG Bind (Kcal/mol)

**1.**	445421	-13,323	-80,94
**2.**	CNP0157012,2	-3,601977097	-46,38411893
**3.**	680292	-5,816534043	-45,66683831
**4.**	76315048	-1,556345045	-42,18639439
**5.**	11376469	-3,905315248	-41,62046264
**6.**	444913	-13,07410674	-41,258579
**7.**	3084228	-5,169753856	-40,59400584
**8.**	4091	-3,267441133	-40,42860935
**9.**	44577078	-3,310492668	-38,88470304
**10.**	441612	-0,478841047	-38,57507864
**11.**	10829011	-4,768576577	-35,22234039
**12.**	CNP0165224,1	-4,305794846	-33,63693605
**13.**	CNP0239276,2	-1,152396691	-33,43290787
**14.**	4091	-0,893512767	-32,01662688
**15.**	108065	-3,639088402	-30,38943501
**16.**	CNP0150614,1	-0,049187762	-30,13016766
**17.**	44577079	-1,1174535	-29,55019194
**18.**	445421	-10,278	-29,02
**19.**	44577080	-7,268040597	-27,9262503
**20.**	4369359	-1,807935785	-27,8746587
**21.**	101392149	-0,066605887	-26,22098209
**22.**	151670	-4,752041381	-25,52987613
**23.**	17697	-0,745436512	-25,18993946
**24.**	65981	-3,92030363	-24,81625686
**25.**	74778	-0,845291945	-23,6746598
**26.**	10438442	-3,02456965	-23,56993282
**27.**	680292	-4,903044669	-23,25020606
**28.**	108065	-7,909653978	-22,86898194
**29.**	21721821	-5,817406934	-22,65298977
**30.**	CNP0165224,1	-1,71115696	-21,65405616
**31.**	2345	-3,765061212	-21,0160504
**32.**	CNP0157012,2	-2,2723215	-20,97958638
**33.**	100418	-4,591609316	-20,52273903
**34.**	3085222	-3,702800207	-19,74592922
**35.**	5284507	-1,410672509	-19,52000981
**36.**	5054	-4,233208157	-18,55598551
**37.**	127149	-1,903374874	-18,31180086
**38.**	CNP0240351,2	-2,032541193	-17,81326595
**39.**	102064358	0,882185438	-17,49681221
**40.**	131753021	-3,262071424	-16,84142161
**41.**	68071	-3,580070486	-16,827953
**42.**	73201	-4,741875239	-16,8260096
**43.**	73447	-4,128478221	-16,62390043
**44.**	5352860	-0,363901551	-16,03475873
**45.**	6483649	-5,651617586	-16,0280744

**46.**	689043	-5,142391786	-15,95833845
**47.**	3084228	-3,7804608	-15,94604546
**48.**	10829011	-1,697273004	-15,80474932
**49.**	240	-4,437166789	-15,65701059
**50.**	6483649	-2,993310071	-15,09661209
**51.**	985	-0,055878854	-14,55748346
**52.**	65981	-3,043296581	-14,19610444
**53.**	4091	-3,147955484	-13,81603479
**54.**	160799	-3,98712768	-13,62894739
**55.**	5280435	-1,839543652	-13,62392191
**56.**	21721823	-4,884804804	-13,19267041
**57.**	12313020	-3,801431579	-13,05685036
**58.**	5318528	-3,813763344	-12,95803157
**59.**	162952798	-4,077555897	-12,73901115
**60.**	6430869	-4,279105709	-12,70501714
**61.**	44577080	-5,660667117	-12,6777736
**62.**	12046149	-4,394120017	-12,51394443
**63.**	12306047	-4,069504557	-12,29435922
**64.**	4369359	-4,201579101	-12,21508636
**65.**	21721823	-6,130855084	-11,74798112
**66.**	18756	-2,515474467	-11,64245978
**67.**	441005	-3,770334775	-11,03058513
**68.**	101420981	-1,042424603	-10,96658056
**69.**	638072	-2,173767561	-10,90337106
**70.**	6432221	-3,858490364	-10,88577399
**71.**	80208	-3,795420954	-10,70762714
**72.**	7463	-4,035442043	-10,48426913
**73.**	10398656	-5,34035526	-10,40976036
**74.**	11230	-5,05060108	-10,35784261
**75.**	151670	-3,694802889	-8,414921234
**76.**	5317570	-3,888495232	-8,297562059
**77.**	100418	-4,297946102	-7,859559179
**78.**	7462	-4,063385849	-7,649568622
**79.**	3085222	-2,966784574	-7,112324136
**80.**	159846	-4,436285892	-6,770130811
**81.**	527113	-3,211130462	-6,359198548
**82.**	10899740	-1,86785712	-5,663449224
**83.**	68071	-1,630587109	-3,935443031
**84.**	73447	-2,773255572	-3,461486896
**85.**	68071	-3,802841988	-3,451629128
**86.**	8794	-4,905435787	-2,481225068
**87.**	92231	-2,123302813	-2,323707691
**88.**	26049	-3,826299071	-2,06447626
**89.**	21721823	-4,808846146	-1,80193531
**90.**	21721823	-3,346648565	-1,106688077

**91.**	91747125	-3,446019959	-1,103719461
**92.**	108065	-6,026078266	-1,072630677
**93.**	17100	-4,2576928	-0,984748715
**94.**	101596917	-1,594076084	-0,662922425
**95.**	22311	-3,897915995	-0,620395559
**96.**	100418	-4,265195077	-0,606524935
**97.**	9548705	-2,982746106	-0,481598961
**98.**	5281516	1,777659622	-0,06416673
**99.**	7461	-4,103850844	0,082550805
**100.**	5281519	-1,50611195	0,145352169
**101.**	21721821	-3,91965708	0,232403328
**102.**	6549	-3,087734716	0,259872163
**103.**	21721821	-3,413936262	0,334284155
**104.**	7460	-3,955529198	0,424006333
**105.**	259846	-1,846539628	0,740299221
**106.**	222284	-1,205057135	1,103610651
**107.**	73201	-0,688158646	1,162905184
**108.**	14759336	-2,647831037	1,326917611
**109.**	10704181	-2,17017652	1,990515575
**110.**	181193	-0,967167634	2,250193103
**111.**	5318528	-2,424994178	2,298651769
**112.**	68071	-3,427693909	2,527385079
**113.**	1742210	-2,476347778	2,827151173
**114.**	5281520	-2,516173817	2,939886856
**115.**	5281855	-1,814479684	3,061144884
**116.**	73447	-4,212803326	3,699751451
**117.**	151670	-2,769331149	3,963782023
**118.**	6483649	-2,294955215	4,605188453
**119.**	93081	-3,630261352	4,822199353
**120.**	108065	-4,718047073	4,855845579
**121.**	31253	-2,834499994	5,704846465
**122.**	101687121	0,901913991	5,754186667
**123.**	68071	-2,069712848	5,955514492
**124.**	3085222	-3,229283081	6,116136731
**125.**	5318528	-4,008706693	6,438294137
**126.**	181193	-4,434906823	7,006789611
**127.**	91753170	-2,960529314	7,057632479
**128.**	13894537	-2,38106562	7,074042408
**129.**	10798255	-3,547439449	7,508897298
**130.**	91753433	-2,957723296	8,35550136
**131.**	12303902	-4,168261165	8,730305194
**132.**	6616	-3,839770528	9,499914406
**133.**	19725	-3,302349498	10,11239909
**134.**	5318528	-3,699817536	10,62116128
**135.**	100418	-5,088774139	10,65625351

**136.**	18756	-3,2740529	11,09858165
**137.**	3085222	-3,908752825	11,32670781
**138.**	2758	-4,732683037	11,33027499
**139.**	5281515	-2,160754767	11,62719811
**140.**	94164	-3,882005405	11,6833335
**141.**	64685	-4,57728833	13,05816606
**142.**	442359	-4,172752396	14,04980923
**143.**	57339298	-2,702218274	14,84346737
**144.**	44577080	-3,932362603	16,16551804
**145.**	3085222	-4,783675572	16,66599046
**146.**	442393	-4,375796087	16,82445968
**147.**	62566	-3,001349898	17,98312558
**148.**	6429080	-4,481777156	19,43956598
**149.**	6918391	-3,652501243	19,7141912
**150.**	21721823	-3,872685749	20,06693002
**151.**	100418	-1,28481162	20,72248088
**152.**	21721821	-4,141272283	22,10511192
**153.**	21721821	-3,46625386	22,85206679
**154.**	181193	-3,400857978	23,89988244
**155.**	92138	-4,212188961	24,8711932
**156.**	5318528	-3,388025309	26,76837287
**157.**	151670	-3,385985884	31,32385038
**158.**	11276107	-4,189227095	34,25928032
**159.**	5281855	-2,475426819	34,69399748
**160.**	5054	-0,7508338	38,29659943
**161.**	73447	-4,061496786	39,39706114
**162.**	689043	-1,810131421	41,16365661
**163.**	689043	-1,162219249	41,66070537
**164.**	5280794	-2,503874285	42,62658519
**165.**	5281855	-0,681188237	55,69992054
**166.**	5281855	-1,534825467	55,70117779

Table S8. Ranking of compounds targeting protein 3K35 based on Binding energy (Kcal/mol)s and MM/GBSA binding energies

Ranking	Pubchem/Coconut ID	Binding energy (Kcal/mol)	MMGBSA dG Bind (Kcal/mol)
**1**	445794	-15,95	-99,88
**2**	14759336	-1,409	-84,63
**3**	11376469	-3,532	-84,47
**4**	101392149	-0,259	-78,59
**5**	44577078	-1,702	-73,34
**6**	10438442	-1,379	-71,1
**7**	44577079	-2,03	-70,08
**8**	127149	-1,983	-69,19

**9**	21721823	-10,815	-68,48
**10**	3085222	-8,073	-64,39
**11**	74778	-1,104	-62,97
**12**	131753021	-0,833	-60,14
**13**	CNP0240351.2	-1,83	-59,68
**14**	73447	-6,967	-59,02
**15**	5280794	-4,719	-58,9
**16**	10829011	-5,208	-57,76
**17**	5281855	-6,419	-57,55
**18**	4369359	-6,668	-56,53
**19**	76315048	-3,11	-56,3
**20**	100418	-6,698	-55,86
**21**	CNP0150614.1	-4,137	-55,84
**22**	5352860	-4,242	-55,2
**23**	21721821	-5,777	-55,07
**24**	441612	2,068	-54,04
**25**	151670	-6,528	-53,13
**26**	5318528	-8,035	-52,22
**27**	5280435	-2,986	-49,99
**28**	CNP0165224.1	-3,352	-49,42
**29**	44577080	-5,045	-49,3
**30**	222284	-4,486	-48,41
**31**	159846	-5,315	-47,39
**32**	73201	-4,399	-47,35
**33**	6483649	-6,271	-47,32
**34**	638072	-1,161	-47,19
**35**	985	-2,458	-46,88
**36**	CNP0239276.2	-1,658	-46,51
**37**	65981	-5,214	-45,81
**38**	2345	-4,844	-45,39
**39**	5284507	-2,167	-44,8
**40**	181193	-4,609	-44,54
**41**	68071	-5,798	-43,85
**42**	101420981	1,567	-43,18
**43**	17697	-0,815	-42,47
**44**	CNP0157012.2	-5,429	-42,15
**45**	3084228	-3,866	-41,85
**46**	162952798	-3,686	-41,28
**47**	689043	-6,885	-41,06
**48**	101687121	-3,066	-38,76
**49**	57339298	-2,975	-37,66
**50**	5281516	2,631	-36,88
**51**	6549	-2,843	-36,77
**52**	12303902	-2,88	-34,75
**53**	6432221	-3,246	-34,7

**54**	6429080	-3,77	-33,69
**55**	442393	-3,066	-33,42
**56**	9548705	-3,188	-33,23
**57**	17100	-3,943	-33,12
**58**	12306047	-3,657	-33,05
**59**	18756	-2,265	-32,83
**60**	5281520	-3,322	-32,74
**61**	91753170	-2,852	-32,54
**62**	8794	-3,888	-32,14
**63**	62566	-2,673	-32,13
**64**	5317570	-3,027	-31,53
**65**	108065	-5,475	-31,33
**66**	160799	-4,245	-31,31
**67**	1742210	-3,442	-31,09
**68**	442359	-3,076	-31,07
**69**	92138	-2,952	-30,83
**70**	10398656	-3,728	-30,8
**71**	6918391	-3,056	-30,63
**72**	527113	-3,388	-30,48
**73**	11230	-3,742	-29,93
**74**	441005	-3,153	-29,7
**75**	93081	-3,103	-29,29
**76**	11276107	-3,643	-28,79
**77**	12313020	-3,585	-28,68
**78**	13894537	-3,265	-28,57
**79**	4091	-3,46	-28,53
**80**	680292	-3,771	-28,37
**80**	31253	-1,554	-28,37
**82**	240	-3,664	-28,31
**83**	91753433	-4,078	-28,17
**84**	5281519	-2,942	-27,96
**85**	7462	-2,885	-27,91
**86**	91747125	-3,251	-27,52
**87**	92231	-3,428	-26,92
**88**	5054	-4,126	-26,23
**89**	10704181	-3,82	-25,88
**90**	80208	-2,09	-25,6
**91**	7461	-2,391	-25,09
**92**	6430869	-3,305	-24,99
**93**	101596917	-3,055	-24,8
**94**	22311	-2,119	-24,46
**95**	10798255	-3,089	-24,18
**96**	5281515	-3,022	-23,95
**97**	7460	-2,892	-23,64
**98**	94164	-2,461	-23,51

**99**	26049	-2,334	-23,39
**100**	19725	-3,318	-22
**101**	7463	-2,755	-20,17
**102**	10899740	0,081	-18,19
**103**	2758	-2,296	-17,49
**104**	12046149	-4,583	-17,23
**105**	6616	-2,188	-16,59
**106**	64685	0,904	41,06
**107**	259846	-3,484	43,15

Table S9. Ranking of compounds targeting protein 2PRG based on Binding energy (Kcal/mol)s and MM/GBSA binding energies

Ranking	Pubchem/Coconut ID	Binding energy (Kcal/mol)	MMGBSA dG Bind (Kcal/mol)
**1.**	11376469	-9,100921415	-79,7321506
**2.**	BRL	-6,788	-72,67
**3.**	151670	-9,718875161	-68,88198909
**4.**	BRL	-10,4	-66,69
**5.**	BRL	-12,06	-65,84
**6.**	445421	-13,24426931	-65,65736827
**7.**	10829011	-6,692215343	-64,67880149
**8.**	3085222	-9,182784549	-63,83537227
**9.**	21721823	-7,144814826	-62,20780325
**10.**	131753021	-8,063610323	-60,76743386
**11.**	44577080	-6,380939735	-59,5544807
**12.**	CNP0165224,1	-4,105046796	-58,66206211
**13.**	44577080	-6,179461591	-58,33282161
**14.**	3084228	-6,920852419	-57,99581104
**15.**	74778	-5,624609566	-57,97412525
**16.**	100418	-6,251267473	-57,79980346
**17.**	5280435	-6,56312641	-57,25250158
**18.**	CNP0165224,1	-6,442751549	-56,74411083
**19.**	76315048	-9,096141199	-56,16990402
**20.**	680292	-5,062842525	-56,00460557
**21.**	638072	-7,530072093	-55,31516068
**22.**	151670	-7,230103196	-54,79211537
**23.**	21721823	-5,362702362	-53,92707378
**24.**	N7F	-5,225518049	-53,86616125
**25.**	127149	-9,837865006	-53,43421447
**26.**	5318528	-5,644978651	-53,40643474
**27.**	10829011	-4,54645186	-52,70806036
**28.**	21721821	-8,58140121	-52,69459836
**29.**	100418	-6,398249179	-52,19702406
**30.**	4369359	-3,942426754	-52,09280378

**31.**	65981	-7,019611282	-51,59923234
**32.**	3085222	-8,1069839	-50,92708842
**33.**	21721823	-9,311629562	-50,55360846
**34.**	3085222	-3,100976425	-49,49477029
**35.**	CNP0157012,2	-3,308336149	-49,48932495
**36.**	17697	-4,339868173	-49,22623597
**37.**	5284507	-3,97817688	-49,12197613
**38.**	73447	-8,774448093	-49,03109274
**39.**	680292	-6,210114889	-48,38143496
**40.**	441612	-8,662448333	-48,28318353
**41.**	181193	-6,299249112	-47,73514991
**42.**	5281516	-4,188155648	-47,63323099
**43.**	2345	-5,333579063	-47,58312053
**44.**	5352860	-5,685784074	-47,35249524
**45.**	73447	-7,175773807	-47,0950032
**46.**	CNP0157012,2	-7,089697606	-47,06342614
**47.**	73201	-6,941531655	-46,7875548
**48.**	445421	-10,27219525	-46,23168115
**49.**	151670	-7,292340703	-45,78653006
**50.**	44577080	-5,011473422	-45,54806586
**51.**	985	-8,408277752	-45,1905179
**52.**	73447	-7,389302002	-44,49361281
**53.**	100418	-5,04120444	-41,73144325
**54.**	65981	-11,30477606	-41,70263677
**55.**	6483649	-5,839349925	-41,08651689
**56.**	10798255	-5,120705862	-40,43597999
**57.**	6483649	-7,266343374	-40,38069158
**58.**	3084228	-7,263023957	-40,25216316
**59.**	5318528	-3,570861721	-39,97106695
**60.**	100418	-8,151650784	-39,86284219
**61.**	68071	-5,93901372	-39,82376329
**62.**	441005	-5,350017313	-39,29081303
**63.**	181193	-7,18588065	-39,08318939
**64.**	21721823	-9,101996001	-38,88303063
**65.**	3085222	-7,120748909	-38,15325353
**66.**	222284	-7,244474068	-38,02938144
**67.**	21721821	-8,225565074	-38,01597807
**68.**	160799	-5,231960906	-37,7823511
**69.**	92231	-5,377338426	-37,71095009
**70.**	6483649	-6,662848853	-37,46070552
**71.**	159846	-7,202208592	-37,30190466
**72.**	80208	-4,077068824	-37,17690591
**73.**	68071	-6,147482791	-36,73175792
**74.**	5318528	-4,498225423	-36,48629169
**75.**	6429080	-4,795919119	-35,36335212
**76.**	5318528	-5,69526155	-35,21136934

**77.**	4369359	-7,246827057	-34,91529101
**78.**	8794	-4,868290366	-34,8872853
**79.**	7463	-4,385216362	-34,88059745
**80.**	10899740	-5,630416932	-34,8311453
**81.**	93081	-5,315966992	-34,21029239
**82.**	18756	-3,284069123	-33,98233224
**83.**	31253	-2,837729367	-33,80938437
**84.**	5281519	-4,051759463	-33,49688427
**85.**	151670	-8,694445193	-33,1333437
**86.**	11230	-4,753668968	-32,96385461
**87.**	7462	-4,349388707	-32,7483724
**88.**	101687121	-4,018889385	-32,61062762
**89.**	442359	-5,18432522	-32,33037661
**90.**	22311	-3,801625161	-32,2232839
**91.**	73201	-3,744432344	-32,19220996
**92.**	18756	-3,091384122	-32,19107749
**93.**	91753433	-6,377194235	-32,06035592
**94.**	5281855	-4,158131176	-32,01535404
**95.**	6549	-3,768297621	-31,84789851
**96.**	9548705	-4,96872032	-31,64234113
**97.**	68071	-4,873373318	-31,52485786
**98.**	21721823	-8,216566994	-31,30990396
**99.**	68071	-5,102472887	-31,11824892
**100.**	7461	-4,20299864	-30,98176532
**101.**	CNP0240351,2	-6,629515366	-30,72352874
**102.**	240	-5,078224251	-30,40957348
**103.**	17100	-5,149485789	-30,40005773
**104.**	5317570	-5,227225398	-30,25153048
**105.**	7460	-4,633658809	-29,94373028
**106.**	5054	-4,896162423	-29,87167468
**107.**	689043	-7,533859828	-29,73372312
**108.**	181193	-6,718770227	-29,70619924
**109.**	442393	-3,800165133	-29,04826325
**110.**	3085222	-6,807120268	-28,80464551
**111.**	21721821	-6,84534131	-28,42813145
**112.**	21721821	-4,875047292	-28,27584867
**113.**	6430869	-5,591153489	-27,38880038
**114.**	12306047	-5,388547664	-27,33292771
**115.**	62566	-5,323704131	-26,64897132
**116.**	6432221	-5,466942712	-26,37953121
**117.**	5281520	-4,235525241	-26,07322134
**118.**	100418	-2,174548652	-25,44621631
**119.**	73447	-7,17424719	-25,2521338
**120.**	91747125	-5,780841051	-25,13963112
**121.**	12313020	-5,262456498	-24,91662294
**122.**	1742210	-4,354550848	-24,68137736

**123.**	6918391	-4,811850548	-24,09877144
**124.**	92138	-5,48612845	-24,04009023
**125.**	26049	-4,240047441	-23,94707601
**126.**	108065	-7,405509783	-23,70104796
**127.**	10398656	-4,968600272	-23,54030097
**128.**	11276107	-5,475368065	-23,12705768
**129.**	101596917	-5,040580385	-23,06412692
**130.**	527113	-5,73222642	-22,95214562
**131.**	94164	-4,658727264	-21,75592105
**132.**	19725	-4,915250181	-21,18119838
**133.**	12303902	-5,248534437	-20,94831014
**134.**	5054	-2,156034201	-20,38744463
**135.**	57339298	-5,079319504	-20,20182231
**136.**	689043	-6,400935027	-19,93042317
**137.**	21721821	-4,536193514	-19,43899769
**138.**	6616	-3,876505905	-19,09355201
**139.**	5281855	-5,896608347	-17,86944239
**140.**	5281855	-3,354532032	-17,84464819
**141.**	2758	-3,046731285	-17,81469904
**142.**	12046149	-5,750915478	-17,74320289
**143.**	13894537	-5,828440764	-17,23938882
**144.**	4091	-0,299427464	-17,17282145
**145.**	4091	-1,677292457	-16,84259535
**146.**	68071	-5,107417295	-16,4793917
**147.**	5318528	-5,472431469	-15,95359117
**148.**	91753170	-5,987193012	-15,24740157
**149.**	5281515	-5,463210868	-14,0214894
**150.**	64685	-3,251709333	-13,8789704
**151.**	5280794	-6,228481025	-13,0538273
**152.**	5281855	-5,436305431	-12,7609116
**153.**	689043	-3,207048481	-11,28056449
**154.**	259846	-4,752285988	-10,60419263
**155.**	10704181	-4,81732691	-10,09789664
**156.**	4091	-1,406212332	-9,66285117
**157.**	108065	-6,386695583	-5,096917896
**158.**	162952798	-5,142326707	-3,694855506
**159.**	108065	-3,042675153	0,860745294

Table S10. Ranking of compounds targeting protein 2Q5S based on Binding energy (Kcal/mol)s and MM/GBSA binding energies

Ranking	Pubchem/Coconut ID	Binding energy (Kcal/mol)	MMGBSA dG Bind (Kcal/mol)
**1.**	151670	-10,75196804	-75,46209911
**2.**	NZA	-10,8	-71,01
**3.**	3085222	-9,244593024	-69,6170643

**4.**	4369359	-9,001152473	-65,50238399
**5.**	21721823	-9,658887805	-60,65936888
**6.**	151670	-8,285733934	-59,97077874
**7.**	5318528	-8,451475424	-58,88760836
**8.**	5352860	-7,278225562	-58,70535917
**9.**	73447	-8,174489257	-58,17227193
**10.**	74778	-5,45969608	-57,48345863
**11.**	4369359	-5,356329851	-56,5820581
**12.**	3084228	-6,693231592	-56,51124909
**13.**	3085222	-7,618265151	-54,60833183
**14.**	3085222	-6,608942809	-54,49904288
**15.**	100418	-8,14378413	-54,4449032
**16.**	21721823	-8,176165878	-54,08709011
**17.**	73447	-9,476063847	-53,93359155
**18.**	10829011	-6,412805753	-53,72421484
**19.**	3085222	-9,868330887	-53,41766751
**20.**	181193	-5,190290148	-52,94101298
**21.**	10398656	-6,645937922	-51,86692049
**22.**	5280435	-6,492079125	-51,34318626
**23.**	5284507	-4,196793236	-51,34167426
**24.**	100418	-7,881146123	-51,16802117
**25.**	151670	-4,807521989	-50,47608723
**26.**	100418	-7,094994973	-50,38566688
**27.**	73201	-9,023452375	-50,36937122
**28.**	5281516	-4,87462303	-49,61100862
**29.**	17697	-3,789640426	-49,4044193
**30.**	151670	-8,084241974	-49,38897147
**31.**	21721821	-7,015559486	-49,20522444
**32.**	10798255	-6,428654094	-49,1789148
**33.**	638072	-3,909777107	-47,55008942
**34.**	5318528	-8,731115302	-47,52680358
**35.**	5281855	-7,329820787	-47,02252428
**36.**	2345	-7,006259192	-46,90480045
**37.**	73447	-8,230961997	-45,97418316
**38.**	73447	-5,21688682	-45,60193069
**39.**	12306047	-6,423819483	-45,42416591
**40.**	101687121	-6,311022489	-44,69687852
**41.**	680292	-6,926655346	-44,47532372
**42.**	100418	-9,694936239	-44,31684753
**43.**	21721821	-7,314936559	-43,31939616
**44.**	680292	-4,046943879	-43,12202824
**45.**	6430869	-6,225336052	-43,0112844
**46.**	527113	-6,115831431	-42,95578251
**47.**	5318528	-4,306797445	-42,88374155
**48.**	68071	-8,150879597	-42,85897908
**49.**	93081	-5,263169769	-42,67522902

**50.**	92231	-6,199921058	-42,32620248
**51.**	442359	-5,212538527	-42,09311031
**52.**	91747125	-5,520344437	-42,05832979
**53.**	5318528	-5,102309331	-41,86775173
**54.**	12046149	-5,77508696	-41,59236287
**55.**	65981	-4,774091917	-41,31169592
**56.**	CNP0165224,1	-5,812005886	-41,30663868
**57.**	6432221	-6,960791464	-40,81018774
**58.**	160799	-6,557682543	-40,79603114
**59.**	6483649	-7,793655961	-40,78642383
**60.**	10899740	-5,512414757	-40,75963727
**61.**	12313020	-6,224690123	-40,60572149
**62.**	159846	-5,767871412	-40,32762994
**63.**	441005	-5,774365676	-40,22164036
**64.**	3084228	-3,736953917	-40,18054162
**65.**	9548705	-5,443590465	-40,07267737
**66.**	13894537	-5,457342588	-40,01881804
**67.**	5317570	-5,756241501	-40,00160699
**68.**	985	-4,368863926	-39,66378479
**69.**	92138	-5,753832963	-39,33407154
**70.**	18756	-4,092905499	-39,30821727
**71.**	12303902	-5,175222643	-39,12163305
**72.**	17100	-5,700701378	-39,03624106
**73.**	21721821	-7,928429533	-39,00919835
**74.**	CNP0157012,2	-6,876587957	-38,76873681
**75.**	6918391	-5,891991845	-38,48460308
**76.**	91753433	-5,763712944	-37,99660593
**77.**	21721821	-7,306168577	-37,58317087
**78.**	19725	-6,515363067	-37,4574287
**79.**	3085222	-5,804976634	-37,19778786
**80.**	31253	-3,615273026	-37,17117244
**81.**	11230	-5,629276478	-37,14630408
**82.**	91753170	-5,923575813	-36,84902764
**83.**	21721821	-4,401489127	-36,75703946
**84.**	62566	-5,692783277	-36,65390906
**85.**	6429080	-5,102908848	-36,63880054
**86.**	442393	-6,237337472	-36,56251437
**87.**	7460	-5,273001997	-36,21558027
**88.**	6549	-4,080845741	-36,02531509
**89.**	80208	-4,75716527	-34,92387575
**90.**	57339298	-6,701384391	-34,84602149
**91.**	18756	-3,828937559	-34,8226791
**92.**	22311	-4,519010422	-34,69741459
**93.**	CNP0165224,1	-3,710664666	-34,68964017
**94.**	5281519	-5,148451391	-34,56610237
**95.**	26049	-4,817847448	-34,45223452

**96.**	7461	-4,998610087	-34,34749439
**97.**	7463	-5,200083479	-34,08902036
**98.**	5281520	-5,862481353	-33,88765539
**99.**	8794	-5,243783296	-33,79232439
**100.**	5318528	-5,760937115	-33,58186527
**101.**	6483649	-5,748927074	-32,98386533
**102.**	6483649	-6,677011978	-32,69839673
**103.**	73201	-1,254734633	-32,52904547
**104.**	1742210	-5,06733121	-31,81354947
**105.**	7462	-4,925822009	-31,67987201
**106.**	68071	-7,391345525	-29,93939649
**107.**	68071	-1,890113228	-29,85778405
**108.**	181193	-6,724140404	-29,81798123
**109.**	10829011	-4,235272855	-29,80959332
**110.**	CNP0157012,2	-5,931207433	-29,43632432
**111.**	5281855	-7,116325598	-29,06817031
**112.**	689043	-5,710647988	-28,71126794
**113.**	65981	-7,67538932	-28,23842628
**114.**	101596917	-5,125252447	-27,29541027
**115.**	240	-5,083523607	-27,04909669
**116.**	94164	-5,721221706	-24,59713025
**117.**	10704181	-5,102995293	-24,59388271
**118.**	68071	-3,013654217	-24,47561892
**119.**	4091	0,043407473	-23,95400931
**120.**	21721823	-6,506223205	-23,56017583
**121.**	5054	-4,226370778	-23,48239266
**122.**	5281515	-4,711238097	-23,46815723
**123.**	162952798	-5,638452533	-22,9454952
**124.**	6616	-3,983003739	-22,55947498
**125.**	2758	-3,794859034	-21,21717262
**126.**	100418	-5,419973474	-20,34515913
**127.**	11276107	-5,684298653	-19,93568555
**128.**	5281855	-7,092251727	-19,04277971
**129.**	44577080	-4,327091781	-16,86053354
**130.**	689043	-3,740766963	-16,50902354
**131.**	4091	-2,349663608	-13,66015993
**132.**	689043	-2,935569211	-13,02026027
**133.**	68071	-4,261624841	-11,28287887
**134.**	64685	-3,779738686	-9,877497768
**135.**	5281855	-2,958494753	-9,745926695
**136.**	4091	-0,744941421	-8,340998716
**137.**	5054	-1,478251797	-5,033750179
**138.**	44577080	-3,238257004	-4,756682346
**139.**	5280794	-2,857338488	0,716900774
**140.**	21721823	-6,487583607	0,880093382
**141.**	44577080	-7,459673842	1,844819741

**142.**	222284	-7,018618891	4,865470947
**143.**	181193	-4,766999657	5,579706403
**144.**	21721823	-9,120330097	37,16563992

Table S11. Ranking of compounds targeting protein 1B2Y based on Binding energy (Kcal/mol)s and MM/GBSA binding energies

Ranking	Pubchem/Coconut ID	Binding energy (Kcal/mol)	MMGBSA dG Bind (Kcal/mol)
**1.**	445421	-13,437	-48,72
**2.**	680292	-5,660370047	-47,21158884
**3.**	CNP0165224,1	-4,840970502	-42,56605954
**4.**	10829011	-6,452031795	-42,48193022
**5.**	44577080	-5,151989549	-41,81744384
**6.**	3084228	-6,596245454	-41,7545377
**7.**	131753021	-3,72006075	-40,66434062
**8.**	444913	-13,50465433	-39,59264028
**9.**	CNP0150614,1	-4,724071339	-38,80607487
**10.**	101392149	-6,270644024	-37,95554805
**11.**	44577079	-5,480012907	-36,59672151
**12.**	222284	-4,544214248	-36,42976284
**13.**	44577080	-4,090530275	-35,44917735
**14.**	74778	-1,465085229	-35,39627357
**15.**	CNP0157012,2	-5,357062677	-35,11200215
**16.**	CNP0239276,2	-4,037613079	-34,70965774
**17.**	108065	-8,676004585	-34,28778644
**18.**	76315048	-5,690014797	-33,06028671

**19.**	4091	-3,397047196	-32,32593053
**20.**	14759336	-4,971378396	-32,32178644
**21.**	6429080	-5,010667443	-32,06659474
**22.**	3085222	-7,326736129	-31,92996351
**23.**	101420981	-1,94940648	-30,64781233
**24.**	151670	-5,995153569	-30,285544
**25.**	10438442	-4,485824974	-30,02080675
**26.**	21721821	-6,057625972	-29,86819269
**27.**	2345	-4,469207485	-29,65785489
**28.**	CNP0240351,2	-3,677848056	-27,65926185
**29.**	11376469	-4,933250363	-27,54891901
**30.**	65981	-4,442825024	-27,5412952
**31.**	12046149	-4,234820187	-27,22900838
**32.**	159846	-5,235231268	-27,04912201
**33.**	44577080	-3,264809243	-26,33670942
**34.**	127149	-5,947535674	-26,03718277
**35.**	445421	-12,345	-25,34
**36.**	92138	-4,017476828	-24,52288552
**37.**	7463	-3,21245911	-24,22560395
**38.**	73447	-4,968225926	-24,0240293
**39.**	4091	-2,369669657	-23,58498886
**40.**	527113	-3,620815166	-23,50535974
**41.**	5280794	-4,643672911	-23,36816044
**42.**	638072	0,351194091	-23,11805063
**43.**	80208	-2,530661595	-23,01385769

**44.**	CNP0157012,2	-4,594750292	-22,9685147
**45.**	CNP0165224,1	-4,525399224	-22,55829641
**46.**	240	-4,522093216	-22,41316737
**47.**	10829011	-3,025422499	-22,23061998
**48.**	10798255	-3,414603405	-21,35029577
**49.**	6483649	-5,765337082	-21,27643262
**50.**	5318528	-3,290962221	-21,2100359
**51.**	21721823	-3,734592362	-21,0186237
**52.**	73201	-5,829395125	-21,00897439
**53.**	5280435	-3,217970775	-20,7691379
**54.**	7461	-3,06561901	-20,47032063
**55.**	3085222	-6,805884845	-20,38988092
**56.**	1742210	-3,570929227	-19,91431931
**57.**	7460	-3,048018399	-19,84390816
**58.**	5281515	-3,234738223	-19,81570887
**59.**	17697	-0,627822054	-19,71632206
**60.**	93081	-3,30137618	-19,44403827
**61.**	680292	-3,499583554	-19,32118649
**62.**	6430869	-3,379650749	-18,73034067
**63.**	102064358	-1,289270594	-18,71792366
**64.**	160799	-3,93061499	-18,1342117
**65.**	3084228	-2,952829599	-17,72942322
**66.**	441005	-3,131686721	-17,27614243
**67.**	18756	-2,360352766	-17,19620188

**68.**	5054	-4,408708781	-17,17023994
**69.**	44577078	-5,398695748	-17,02251366
**70.**	5281520	-3,24413849	-16,88124524
**71.**	21721823	-2,823774309	-16,84693141
**72.**	10398656	-3,808084116	-16,83987636
**73.**	9548705	-3,418644765	-16,57271987
**74.**	13894537	-3,726124828	-16,57095952
**75.**	8794	-3,475348705	-16,41788123
**76.**	68071	-3,478006328	-16,2240499
**77.**	22311	-2,883998732	-16,1571426
**78.**	442359	-3,214010574	-16,08600728
**79.**	441612	-5,722020847	-15,96511396
**80.**	108065	-5,108918508	-15,95422682
**81.**	4369359	-3,815241388	-15,90066437
**82.**	12306047	-3,335438937	-15,85860904
**83.**	94164	-3,169198841	-15,60060054
**84.**	11230	-5,162075534	-15,24851149
**85.**	181193	-0,643322055	-15,0488796
**86.**	4091	-3,744172711	-14,42165122
**87.**	12303902	-3,28019621	-14,16325577
**88.**	11276107	-3,560515158	-13,84135512
**89.**	26049	-3,08140801	-13,37864622
**90.**	91747125	-3,127735485	-13,29540606
**91.**	62566	-2,90229727	-12,7402396
**92.**	6918391	-2,979940169	-12,51833735

**93.**	6432221	-4,063056948	-12,47211339
**94.**	2758	-2,470085344	-12,44336639
**95.**	5284507	-1,943162082	-12,40213545
**96.**	18756	-1,774096452	-12,34526923
**97.**	6616	-2,636449743	-12,19348748
**98.**	5281519	-2,941745806	-11,89138099
**99.**	101596917	-3,115935435	-11,83267371
**100.**	5317570	-3,277916697	-11,57845632
**101.**	442393	-3,263926833	-11,50255943
**102.**	10704181	-3,222664933	-11,45506786
**103.**	101687121	3,184731131	-11,28039823
**104.**	68071	-4,417191162	-11,24363065
**105.**	162952798	-3,939862378	-10,85469803
**106.**	100418	-4,997622712	-10,404317
**107.**	5318528	-1,615580799	-10,38602306
**108.**	91753170	-3,596885536	-10,1893926
**109.**	5352860	-1,319372111	-9,59988017
**110.**	10899740	-3,59653264	-9,527398471
**111.**	3085222	-3,048370388	-9,449828936
**112.**	689043	-4,815077972	-9,081571399
**113.**	5281516	-1,407820133	-8,944241404
**114.**	108065	-6,077494479	-8,154323127
**115.**	7462	-2,812145254	-7,519669013
**116.**	259846	-3,63463095	-7,35535098

**117.**	12313020	-3,228409376	-7,323561813
**118.**	57339298	-3,394589332	-6,293855017
**119.**	3085222	-3,283946587	-6,100157785
**120.**	17100	-3,054483301	-5,762379325
**121.**	985	-0,0964129	-5,74624213
**122.**	3085222	-4,915135195	-5,233450234
**123.**	5318528	-3,625526149	-5,137263819
**124.**	91753433	-2,929801689	-5,111256129
**125.**	19725	-3,240343018	-4,801702334
**126.**	6549	-2,583360324	-4,500531601
**127.**	31253	-1,122122047	-3,893970281
**128.**	108065	-5,128755766	-3,3987086
**129.**	21721821	-3,859099908	-3,166082733
**130.**	5281855	-5,280785814	-0,92785974
**131.**	92231	-3,451112807	-0,719605321
**132.**	100418	-4,638501163	-0,67251418
**133.**	73201	1,606133324	-0,442778483
**134.**	21721821	-4,622094206	-0,375788209
**135.**	317	-2,853589338	-0,333779429
**136.**	5318528	-3,525802562	-0,091964154
**137.**	21721821	-4,251081829	-0,08248297
**138.**	64685	-2,74504232	0,266550557
**139.**	5281855	-4,167584764	4,334310812
**140.**	5054	-0,998757803	4,801354875

**141.**	151670	-5,058126854	5,048225187
**142.**	73447	-3,989133991	5,066258957
**143.**	21721821	-5,295539263	5,170904109
**144.**	181193	-2,274815023	5,838311705
**145.**	6483649	-3,513955903	6,041996078
**146.**	100418	-3,1178471	6,161423624
**147.**	73447	-5,099764288	6,218594235
**148.**	21721823	-1,187486046	7,066097858
**149.**	65981	-2,344034766	7,954588334
**150.**	68071	-2,999996184	9,702873707
**151.**	73447	-0,656140653	10,2233831
**152.**	151670	-3,584309813	13,16209437
**153.**	100418	-2,348953862	13,26328474
**154.**	5281855	-3,765845235	15,08795276
**155.**	68071	0,002137776	16,1021294
**156.**	21721823	-1,678808287	17,77917537
**157.**	5281855	-1,733975962	18,35963621
**158.**	151670	-2,134467814	23,08921109
**159.**	181193	-3,388832672	25,28556531
**160.**	68071	-3,768103696	25,87963941
**161.**	5318528	-3,12990576	28,13984164
**162.**	100418	-2,463554988	30,00185458
**163.**	6483649	-1,966604716	31,12227719
**164.**	21721823	-1,291311984	41,61893105

**165.**	689043	-<br>2,657286595	48,23917621
**166.**	689043	-<br>1,229905752	62,66069845

Table S12. Ranking of compounds targeting protein 2QBQ based on Binding energy (Kcal/mol)s and MM/GBSA binding energies

Ranking	Pubchem/Coconut ID	Binding energy (Kcal/mol)	MMGBSA dG Bind (Kcal/mol)
**1.**	17759043	-9,28	-72,96
**2.**	14759336	-2,991	-71,95
**3.**	21721823	-5,222	-63,62
**4.**	441612	-3,061	-62,69
**5.**	101420981	0,657	-61,43
**6.**	181193	-4,79	-54,8
**7.**	11376469	-3,297	-52,49
**8.**	44577078	-2,627	-51,61
**9.**	5281855	-5,825	-51,07
**10.**	CNP0150614.1	-2,943	-50,45
**11.**	10438442	-2,445	-50,29
**12.**	101392149	-2,983	-49,87
**13.**	638072	-2,224	-49,2
**14.**	3085222	-4,288	-46,94
**15.**	680292	-3,719	-46,15
**16.**	131753021	0,604	-46,12
**17.**	CNP0157012.2	-4,533	-46,04
**18.**	5280435	-1,407	-45,24
**19.**	100418	-3,836	-45,19
**20.**	10829011	-4,058	-44,93
**21.**	44577080	-4,182	-44,81
**22.**	CNP0240351.2	-3,628	-44,33
**23.**	21721821	-5,976	-43,57
**24.**	CNP0165224.1	-3,388	-42,38
**25.**	CNP0239276.2	-2,233	-42,25
**26.**	73201	-4,509	-41,82
**27.**	74778	2,209	-41,82
**28.**	76315048	-2,566	-41,52
**29.**	17697	-1,336	-41,04
**30.**	985	-2,512	-40,86
**31.**	151670	-4,75	-40,72
**32.**	68071	-4,625	-39,99
**33.**	73447	-4,294	-39,22

**34.**	65981	-5,278	-39,21
**35.**	689043	-6,755	-38,83
**36.**	4369359	-3,46	-37,77
**37.**	160799	-4,391	-36,88
**38.**	3084228	-3,747	-36,88
**39.**	127149	-1,593	-36,81
**40.**	5280794	-3,432	-36,57
**41.**	222284	-3,152	-36,3
**42.**	5352860	-2,033	-35,88
**43.**	444913	-9,873	-35,86
**44.**	162952798	-4,157	-34,96
**45.**	92138	-3,371	-33,61
**46.**	8794	-3,669	-33,22
**47.**	108065	-4,932	-33,18
**48.**	91753433	-3,048	-32,19
**49.**	527113	-2,936	-32,1
**50.**	94164	-2,194	-31,86
**51.**	2345	-2,496	-30,95
**52.**	91753170	-3,069	-30,17
**53.**	159846	-4,622	-29,68
**54.**	9548705	-2,178	-28,9
**55.**	5318528	-4,157	-28,61
**56.**	12306047	-2,758	-27,94
**57.**	10398656	-3,159	-27,85
**58.**	6429080	-2,956	-27,77
**59.**	10798255	-2,704	-27,55
**60.**	101687121	-2,301	-26,98
**61.**	12313020	-2,345	-26,82
**62.**	7462	-2,442	-26,79
**63.**	31253	-1,009	-26,79
**64.**	5317570	-3,003	-26,58
**65.**	240	-3,575	-26,53
**66.**	7460	-2,581	-26,47
**67.**	26049	-2,744	-26,46
**68.**	4091	-2,284	-26,21
**69.**	5281515	-2,134	-26,13
**70.**	11276107	-2,992	-26,03
**71.**	10704181	-2,406	-26,01
**72.**	7461	-2,757	-25,68
**73.**	93081	-3,035	-25,52
**74.**	6549	-2,316	-25,47
**75.**	6483649	-4,08	-25,34
**76.**	259846	-1,864	-25,33
**77.**	5281516	-0,734	-25,32
**78.**	7463	-2,406	-25,22

**79.**	5284507	-2,307	-25,1
**80.**	442393	-3,034	-24,94
**81.**	101596917	-2,077	-24,92
**82.**	442359	-3,022	-24,9
**83.**	80208	-2,994	-24,85
**84.**	5281519	-2,3	-24,4
**85.**	13894537	-2,136	-24,34
**86.**	11230	-2,416	-22,92
**87.**	92231	-2,32	-22,78
**88.**	441005	-2,84	-22,7
**89.**	1742210	-2,62	-22,44
**90.**	64685	-2,136	-21,99
**91.**	12303902	-2,854	-21,95
**92.**	57339298	-2,95	-21,55
**93.**	62566	-2,921	-21,32
**94.**	91747125	-2,861	-21,16
**95.**	5054	-2,574	-20,44
**96.**	17100	-3,073	-20,11
**97.**	6432221	-2,694	-20,03
**98.**	22311	-2,297	-19,98
**99.**	2758	-2,262	-19,79
**100.**	18756	-1,489	-19,59
**101.**	5281520	-2,415	-18,55
**102.**	10899740	-2,619	-17,72
**103.**	6430869	-2,779	-16,97
**104.**	12046149	-2,965	-14,9
**105.**	19725	-2,255	-12,11
**106.**	6918391	-2,34	-9,3
